# Comprehensive genomic characterization of multidrug-resistant *Klebsiella pneumoniae* from North India

**DOI:** 10.3389/fmicb.2026.1831365

**Published:** 2026-09-24

**Authors:** Nargis Kaur Bali, Aktarul Islam Siddique, Umaya Majid, Dona Mathew, Susheem Ramakrishnan, Biswajyoti Borkakoty

**Affiliations:** 1 Sher-i-Kashmir Institute of Medical Sciences, Srinagar, India; 2 Regional Medical Research Centre (ICMR), Dibrugarh, India

**Keywords:** blaOXA-232, carbapenem resistance, *Klebsiella pneumoniae*, MLST, phylogenomics, resistome, whole-genome sequencing

## Abstract

**Background:**

*Klebsiella pneumoniae* is a major cause of healthcare-associated infections and an important contributor to the global burden of antimicrobial resistance. However, comprehensive genomic epidemiological data on multidrug-resistant (MDR) *K. pneumoniae* from North India remain limited.

**Methods:**

Ninety-six non-duplicate clinical *K. pneumoniae* isolates collected from a tertiary-care hospital in North India during 2022–2024 underwent antimicrobial susceptibility testing and *de novo* whole-genome sequencing (WGS). Following genomic quality assessment and targeted re-evaluation, 90 genomes were retained for genome-based analyses. MLST and pangenome analyses included 72 and 69 genomes, respectively, according to their respective inclusion criteria. Genome-based analyses included capsular typing, resistome and virulome profiling, plasmid replicon prediction, pangenome analysis, identification of resistance-associated chromosomal mutations, and phylogenomic reconstruction.

**Results:**

Phenotypic analysis showed carbapenem resistance in 93/96 (96.9%) isolates. Genomic analysis identified 32 sequence types, with ST515 (37.5%; 27/72) as the predominant lineage, while KL62 (60.0%; 54/90) was the most common capsular locus. The predominant carbapenemase gene was *bla*_OXA-232_ (75.6%; 68/90), followed by *bla*_NDM-1_ (14.4%; 13/90) and *bla*_NDM-5_ (8.9%; 8/90). A total of 82 antimicrobial resistance genes spanning 16 antibiotic classes were identified, with *fosA* (86/90, 95.6%), *oqxA* (85/90, 94.4%), *qnrS1* (75/90, 83.3%), and *bla*_CTX-M-15_ (69/90, 76.7%) being the most prevalent. Pangenome analysis of 69 genomes identified 13,720 gene families, indicating extensive accessory genome diversity. Resistance-associated mutations involving *ompK35*, *ompK36*, *gyrA*, *parC*, *ramR*, and *cirA* were identified. Virulence profiling revealed conserved genes for adherence, biofilm formation, secretion systems, and iron acquisition, whereas *rmpA* and *rmpA2* were absent. Phylogenomic analysis demonstrated clustering according to sequence type and capsular locus.

**Conclusion:**

This study provides a comprehensive genomic characterization of MDR *K. pneumoniae* circulating in a North Indian tertiary care hospital. The predominance of the ST515 and KL62 lineage, together with high prevalence of *bla*_OXA-232_, with an extensive accessory genome and diverse resistance determinants, highlights the value of WGS for genomic surveillance of carbapenem-resistant *K. pneumoniae* and for informing infection prevention and antimicrobial stewardship.

## 1 Introduction

*Klebsiella pneumoniae* (*K. pneumoniae*) is a Gram-negative opportunistic pathogen responsible for a wide range of healthcare- and community-acquired infections, including pneumonia, urinary tract infections, bloodstream infections, and wound infections (Russo and Marr, 2019; Chang et al., 2021). Its clinical significance has increased globally owing to the rapid emergence of multidrug-resistant (MDR) strains, which severely limit therapeutic options and are associated with increased morbidity and mortality (Logan and Weinstein, 2017; Wyres et al., 2020). Carbapenem-resistant *K. pneumoniae* (CRKP) has been designated a critical priority pathogen by the World Health Organization because of its widespread dissemination and major threat to public health (WHO, 2024). Carbapenem resistance arises through a combination of acquired and chromosomal mechanisms, including production of carbapenemases (e.g., *bla*_NDM_ and *bla*_OXA-48-_like), extended-spectrum β-lactamases (ESBL) determinants such as *bla*_CTX-M_, and certain *bla*_SHV_, and *bla*_TEM_ variants acting in conjunction with porin alterations, as well as multidrug efflux systems (Pitout et al., 2015; Logan and Weinstein, 2017). The continued emergence and global dissemination of carbapenemase-producing high-risk clonal lineages have further accelerated the burden of antimicrobial resistance (Lee et al., 2016; Wyres et al., 2020).

In India and other parts of South Asia, *K. pneumoniae* has emerged as a leading cause of healthcare-associated infections, with increasing prevalence of carbapenem-resistant isolates carrying OXA-48-like and NDM carbapenemases and the expansion of high-risk clonal lineages such as ST231 and ST11 (Veeraraghavan et al., 2017; David et al., 2019; Shankar et al., 2019). Although high-risk lineages such as ST231 predominate in most Indian genomic studies, other sequence types, including ST515, have also been reported among clinical *K. pneumoniae* isolates, suggesting ongoing diversification of multidrug-resistant lineages within the country (Shamanna et al., 2024). Recent whole-genome sequencing studies have further demonstrated extensive genomic diversity among Indian *K. pneumoniae* isolates, revealing considerable sequence type and capsular diversity together with the coexistence of antimicrobial resistance and virulence determinants (Raj et al., 2022; Shamanna et al., 2024; Sharma et al., 2024). Notably, ST231 has consistently emerged as one of the predominant carbapenem-resistant lineages in India, frequently associated with OXA-48-like carbapenemases and clinically important siderophore systems, underscoring its significance as a high-risk clone (Shankar et al., 2019; Shamanna et al., 2024; Sharma et al., 2024).

In addition to acquired resistance genes, chromosomal alterations involving outer membrane porins (*ompK35* and *ompK36*) and multidrug efflux systems may further contribute to antimicrobial resistance in *K. pneumoniae* (Becerra-Aparicio et al., 2025; Ullah S et al., 2024). Despite recent advances, comprehensive genomic epidemiological data from North India remain limited, particularly studies integrating multilocus sequence typing (MLST), resistome, virulome, plasmid, and phylogenomic analyses across diverse clinical specimens. Whole-genome sequencing (WGS) has emerged as a powerful tool for high-resolution characterization of *K. pneumoniae*, enabling simultaneous analysis of population structure, sequence types, capsular loci, antimicrobial resistance determinants, virulence factors, plasmid content, pangenome diversity and phylogenetic relationships, thereby facilitating genomic surveillance of high-risk multidrug-resistant lineages (Holt et al., 2015; Wyres et al., 2020; Jiang et al., 2025).

Although several genomic studies have characterized bloodstream and urinary isolates, comprehensive genomic analyses of *K. pneumoniae* recovered from diverse clinical specimens remain limited in North India. In this context, the present study employed whole-genome sequencing to comprehensively characterize clinical *K. pneumoniae* isolates from a tertiary care hospital, integrating sequence typing, capsular typing, pangenome analysis, resistome and virulome profiling, plasmid prediction, resistance-associated chromosomal mutations, and phylogenomic analysis. These findings provide valuable insights into the genomic epidemiology and antimicrobial resistance landscape of multidrug-resistant *K. pneumoniae* circulating in the study population.

## 2 Materials and methods

### 2.1 Study setting and clinical isolates

This study was conducted using clinical specimens collected between 2022 and 2024 at the Sher-i-Kashmir Institute of Medical Sciences (SKIMS), Srinagar, Jammu & Kashmir, India, a tertiary-care referral hospital. Specimens were received through routine diagnostic microbiology services from both inpatient and outpatient departments, including the Intensive Care Unit (ICU), Neonatal Intensive Care Unit (NICU), Surgical Intensive Care Unit (SICU), and other specialized clinical units. Sample types included blood, respiratory specimens, urine, pus, wound swabs, and other clinically relevant specimens.

The study included 96 non-duplicate carbapenem-resistant *K. pneumoniae* isolates recovered during the study period, selected for whole-genome sequencing using a systematic sampling strategy in which approximately every alternate eligible isolate was included. Clinical and phenotypic analyses were performed on all 96 isolates. Of the 96 isolates, 95 yielded genomes meeting the initial sequencing quality-control criteria; following re-evaluation of genomes lacking *bla*_SHV_ or harboring *bla*_ACT_, five genomes were excluded from downstream genomic analyses, resulting in 90 genomes retained for genome-based analyses. MLST analysis was performed on 72 genomes with complete allelic profiles for all seven housekeeping genes, while pangenome analysis included 69 genomes meeting the predefined criteria of ≥90% completeness and <7% contamination.

### 2.2 Bacterial isolation, identification, and antimicrobial susceptibility testing

All clinical specimens were processed following standard microbiological protocols. Presumptive identification of *Klebsiella pneumoniae* (*K. pneumoniae*) isolates relied on colony morphology, Gram-stain characteristics, and conventional biochemical reactions, with species-level confirmation subsequently obtained using the VITEK® 2 Compact automated identification system (bioMérieux, France).

Antimicrobial susceptibility testing (AST) was performed through a combination of conventional methods and the VITEK® 2 Compact system, with results interpreted per the Clinical and Laboratory Standards Institute (CLSI) breakpoints current at the time of testing. Individual antibiograms were generated for each isolate to enable phenotypic resistance profiling.

### 2.3 DNA extraction and whole-genome sequencing

Genomic DNA was isolated from pure *K. pneumoniae* cultures using the QIAamp DNA Mini Kit (Qiagen, USA) according to the manufacturer’s protocol. The concentration and purity of the extracted DNA were subsequently measured using a Qubit 4.0 fluorometer (Invitrogen, Carlsbad, CA, USA).

Whole-genome sequencing was performed by Eurofins Genomics India Pvt. Ltd. and carried out on an Illumina platform, generating 150 bp paired-end reads.

### 2.4 Bioinformatic workflow

Raw paired-end reads underwent quality assessment using FastQC and adapter trimming with Trimmomatic. High-quality reads were assembled *de novo* using Unicycler, and the resulting genome assemblies were subsequently used for genome annotation (Prokka), MLST and K-locus assignment (Kleborate), AMR gene identification (AMRFinderPlus), plasmid replicon detection (PlasmidFinder), virulence factor profiling (VFDB), pangenome analysis (Roary), and core genome SNP phylogenetic reconstruction (IQ-TREE).

### 2.5 Quality control and genome assembly

Read quality was first assessed with FastQC (Babraham Bioinformatics) under default settings. Trimmomatic v0.39 (Bolger et al., 2014) was then used to remove adapter sequences, reads with ambiguous base content exceeding 5% N, and low-quality bases, defined as reads containing more than 10% of positions with a Phred score below 25.

*De novo* genome assembly and error correction were performed with Unicycler v0.5.1 (Wick et al., 2017) under default parameters. One assembly failed the predefined genome quality control criteria and was excluded from all downstream genomic analyses.

### 2.6 Genome analysis, sequence typing, and resistome profiling

Kleborate v2.3.1 (Lam et al., 2021) was used to perform multilocus sequence typing (MLST) and capsular (K-type) characterization of the isolates. The MLST distribution among the *K. pneumoniae* isolates was visualized as a minimum spanning tree constructed with GrapeTree v1.5.0, which implements the MSTreeV2 algorithm, using the associated online platform^1^ (Zhou et al., 2018). MLST analysis was restricted to genomes with complete and high-confidence allelic profiles for all seven housekeeping genes.

Genome assemblies were further scaffolded through reference-guided scaffolding using Ragout v2.3 (Kolmogorov et al., 2018), with *K. pneumoniae* strain HS11286 (accession number NC_016845.1) as the guide genome. Kleborate was subsequently re-run on these reference-guided assemblies for isolates that lacked complete allelic profiles at one or more housekeeping loci, in order to improve MLST data recovery. The Ragout-scaffolded assemblies were used exclusively for recovery of incomplete MLST allelic profiles and were not used for antimicrobial resistance, virulence, plasmid, pangenome, or phylogenomic analyses. Genomes with incomplete or ambiguous allele assignments resulting from fragmented assemblies were excluded from MLST-based analyses, yielding 72 genomes for sequence type determination.

AMRFinderPlus v4.0.23 (Feldgarden et al., 2021), with the NCBI reference database, was applied to the original *de novo* genome assemblies of the 90 genomes retained for downstream genomic analyses to identify antimicrobial resistance genes and resistance-associated point mutations (RAMP). Virulence factor genes were screened from the original *de novo* assemblies using the Virulence Factor Database (VFDB), web-based tool, drawing on the complete Virulence Factor Database (Liu et al., 2022). The distribution of AMR genes, virulence factors, and MLST types across the isolates was summarized in a heatmap generated in R using the pheatmap v1.0.12 package. A matrix plot, generated in R with the ggplot2 v3.4.4 package, was used to compare the distribution of resistance-associated point mutations between carbapenem-resistant and non-carbapenem-resistant isolates. The distribution of AMR genes across hospital wards, corresponding to the source of sample collection, was further illustrated using an UpSet-style plot built in R with ggplot2 v3.4.4.

Plasmid replicons were identified from the original *de novo* assembliesusing PlasmidFinder v2.1.6 with its locally installed reference database^2^ (Carattoli et al., 2014). Relationships between representative carbapenem-resistant and carbapenem-susceptible isolates and their plasmid replicons were visualized using Sankey diagrams generated in SankeyMATIC^3^.

### 2.7 Annotation, pangenome, and phylogenetic analysis

The original *de novo* genome assemblies were annotated using Prokka v1.14.6 (Seemann, 2014). Pangenome analysis of the annotated genomes was subsequently performed using Roary v3.13.0 (Page et al., 2015). Pangenome genes were classified as core (99–100%), soft-core (95–99%), shell (15–95%), or cloud (<15%), according to Rocha et al. (2022). Genome assembly quality, completeness, and contamination were assessed using QUAST v5.3.0 and CheckM2 v1.1.0. Assembly metrics for all 95 sequenced genomes are provided in Supplementary Table 1.

Core genome SNP analysis was performed using the original *de novo* genome assemblies. Reference genomes were selected to provide global phylogenetic context by representing major internationally distributed sequence types across multiple geographic regions and were not intended to infer direct evolutionary relationships. The final phylogenetic dataset comprised 133 *K. pneumoniae* genomes, including 69 study isolates, 63 publicly available genomes selected from the Institut Pasteur *Klebsiella pneumoniae* database to represent major sequence types across five continents and provide global phylogenetic context (Supplementary Table 2), and the reference strain *K. pneumoniae* HS11286 (GenBank accession no. NC_016845.1). Core genome alignment of these 133 genomes spanned 2,229,083 bp, from which 41,268 core-genome SNPs (cgSNPs) were extracted using SNP-sites v2.5.1 (Page et al., 2016) for phylogenetic reconstruction. A maximum-likelihood (ML) phylogenetic tree was inferred from the cgSNP alignment using IQ-TREE v3.0.1 (Wong et al., 2026) and subsequently visualized in iTOL v7 (Letunic and Bork, 2024).

### 2.8 Data availability

The whole-genome sequencing data generated in this study have been deposited in the NCBI under BioProject accession PRJNA1392785. The assembled genomes are available in the GenBank database under accession numbers NZ_CM153146.1–NZ_CM153188.1 and NZ_CM153200.1–NZ_CM153250.1. The corresponding raw sequencing reads have been deposited in the NCBI Sequence Read Archive (SRA) under accession numbers SRR38783703–SRR38783797, with associated BioSample accession numbers SAMN54271601–SAMN54271695.

### 2.9 Ethical approval

The clinical samples used in this study were originally collected as part of routine diagnostic procedures at Sher-i-Kashmir Institute of Medical Sciences (SKIMS), Srinagar. For the purpose of whole-genome sequencing and subsequent genomic analyses, bacterial isolates were used in an anonymized manner, with only sample identifiers and limited clinical metadata (e.g., specimen source) retained. The study protocol was reviewed and approved by the Institutional Ethics Committee of ICMR–Regional Medical Research Centre, North East (ICMR-RMRC NE), Dibrugarh (Approval No. RMRC/Dib/IEC (Human)/2024–25/257 dated 04/06/2024).

## 3 Results

### 3.1 Clinical antibiotic susceptibility characteristics of *K. pneumoniae* isolates

Figure 1 summarizes the clinical profiles and antimicrobial resistance phenotypes of the *K. pneumoniae* isolates. Among the 96 clinical isolates, 93 (96.9%) were phenotypically resistant to carbapenems. Resistance to meropenem and imipenem was observed in 92 and 93 isolates, respectively, with one isolate remaining susceptible to meropenem despite resistance to imipenem. All isolates were resistant to ciprofloxacin, amikacin, gentamicin, levofloxacin, and co-trimoxazole. Resistance to cefepime, ceftriaxone, and cefotaxime was detected in 95 isolates, whereas 93 and 92 isolates were resistant to ceftazidime-avibactam and piperacillin-tazobactam, respectively. Only one isolate exhibited resistance to tigecycline (Supplementary Table 3). Among the source populations, inpatient department (IPD) cases accounted for the largest proportion of isolates (38.54%, 37/96), followed by outpatient department (OPD) cases (26.04%, 25/96).

**Figure 1:**
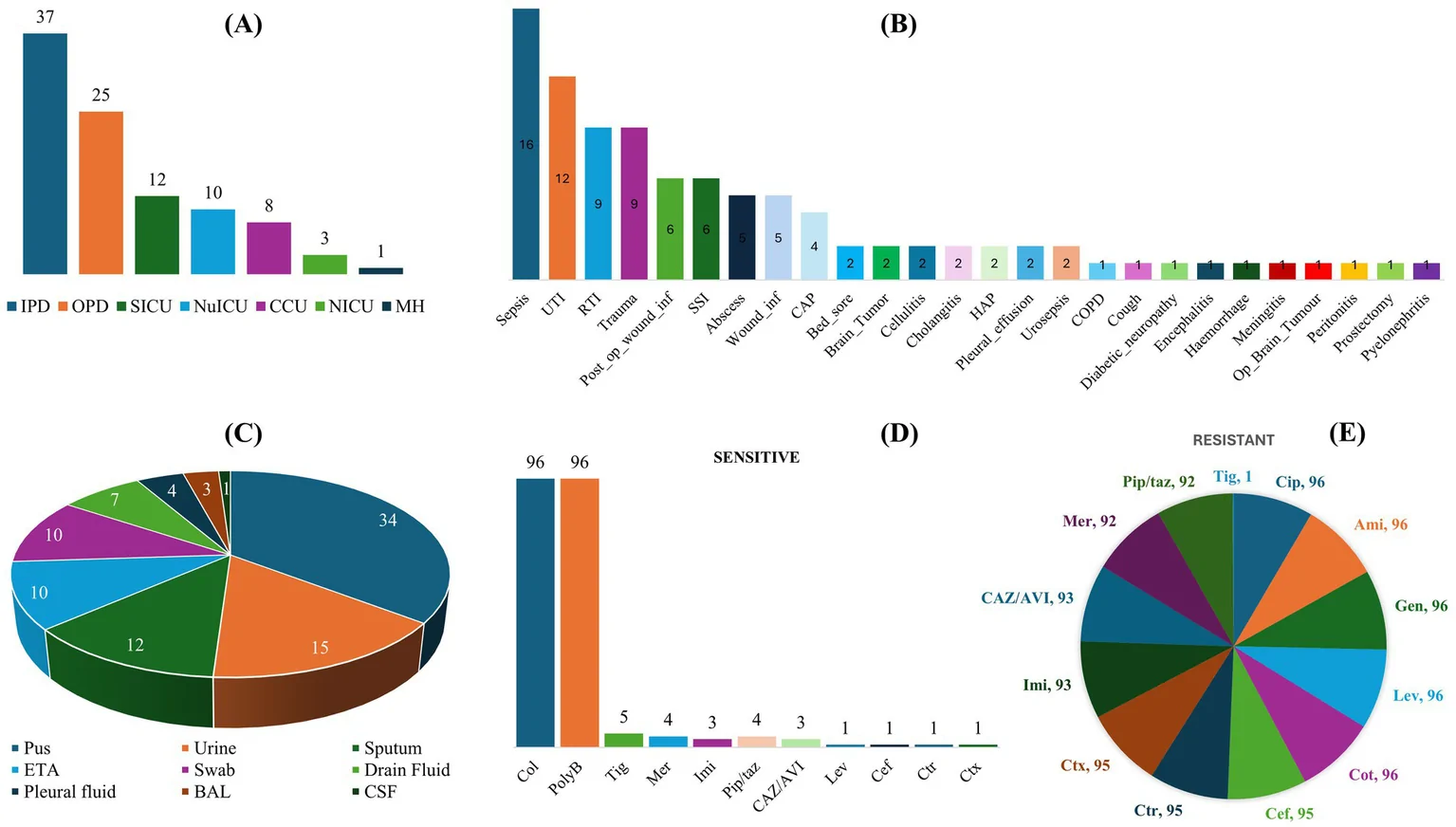
Clinical characteristics and antimicrobial susceptibility profile of *K. pneumoniae* isolates (*n* = 96). **(A)** Distribution of isolates by hospital department. **(B)** Clinical diagnoses associated with *K. pneumoniae* infections. **(C)** Distribution of isolates according to specimen type. **(D)** Antimicrobial susceptibility pattern of the isolates. **(E)** Antimicrobial resistance profile showing the number of resistant isolates for each antibiotic.

### 3.2 MLST distribution and capsular typing

Of the 90 genomes retained for downstream genomic analyses, 72 yielded complete allelic profiles for all seven MLST housekeeping genes and were included in MLST analysis. The remaining genomes contained incomplete or fragmented housekeeping loci resulting from lower assembly contiguity and were therefore excluded from sequence type assignment. The MLST-based minimum spanning tree demonstrated substantial genetic diversity, with 32 sequence types identified among the 72 isolates assigned complete allelic profiles (Figure 2). ST515 was the predominant lineage (37.5%; 27/72), followed by ST17 (13.9%; 10/72). The remaining sequence types were represented by one or a few isolates, indicating considerable lineage diversity within the study collection. The star-like topology centered on the dominant STs is consistent with clonal expansion accompanied by limited allelic diversification, occurring alongside the coexistence of numerous minor lineages.

**Figure 2:**
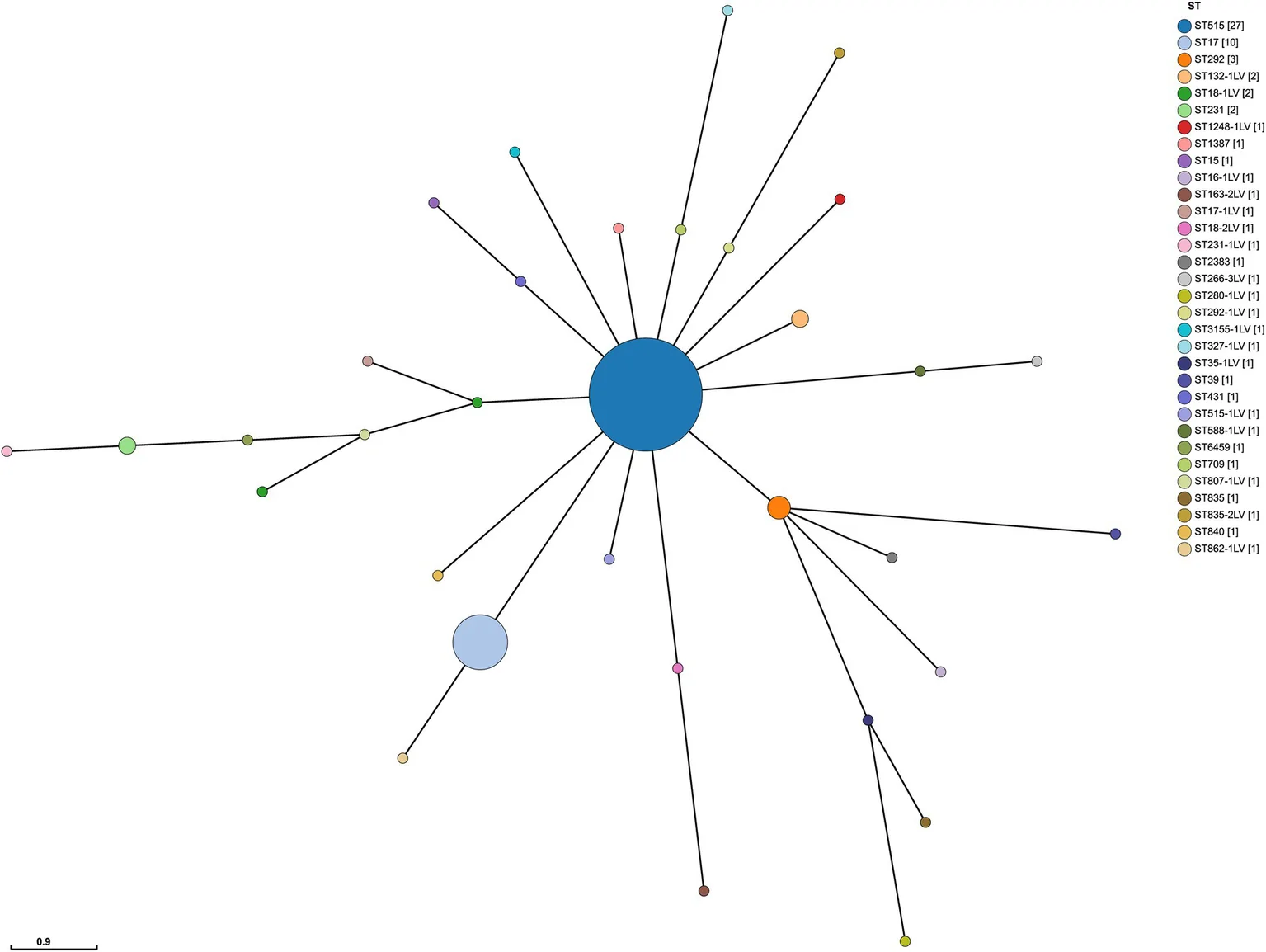
Grape tree representation of multilocus sequence typing (MLST) profiles of *Klebsiella pneumoniae* isolates (*n* = 72). Each node represents a unique sequence type (ST), with node size proportional to the number of isolates belonging to that ST. Colors indicate different STs, as shown in the legend.

Capsular typing identified nine distinct K-loci among the isolates. KL62 was the most common K-locus (60.0%; 54/90), followed by KL51 (13.3%; 12/90) and KL25 (10.0%; 9/90).

### 3.3 Pangenomic analysis

Pangenome analysis of the 69 high-quality genomes that met the predefined completeness (≥90%) and contamination (<7%) thresholds, identified 13,720 gene families, comprising 1,574 core genes, 2,069 soft-core genes, 3,307 shell genes, and 6,770 cloud genes, indicating extensive accessory genome diversity and substantial genomic heterogeneity among the study isolates.

### 3.4 Antibiotic resistance genes

Antimicrobial resistance (AMR) genes identified by AMRFinderPlus were mapped across the MLST-defined isolates and visualized together with virulence factor genes (Figure 3). Analysis of the 90 genomes identified 82 AMR genes belonging to 16 antibiotic classes, including aminoglycosides, β-lactams, bleomycin, fosfomycin, macrolides, phenicols, quinolones, rifamycins, sulfonamides, tetracyclines, and trimethoprim. The fosfomycin resistance gene *fosA* was the most prevalent determinant, detected in 86/90 (95.6%) isolates, followed by *oqxA* in 85/90 (94.4%), and *qnrS1* in 75/90 (83.3%), while *bla*_CTX-M-15_ was the predominant ESBL gene in 69/90 (76.7%) isolates.

**Figure 3:**
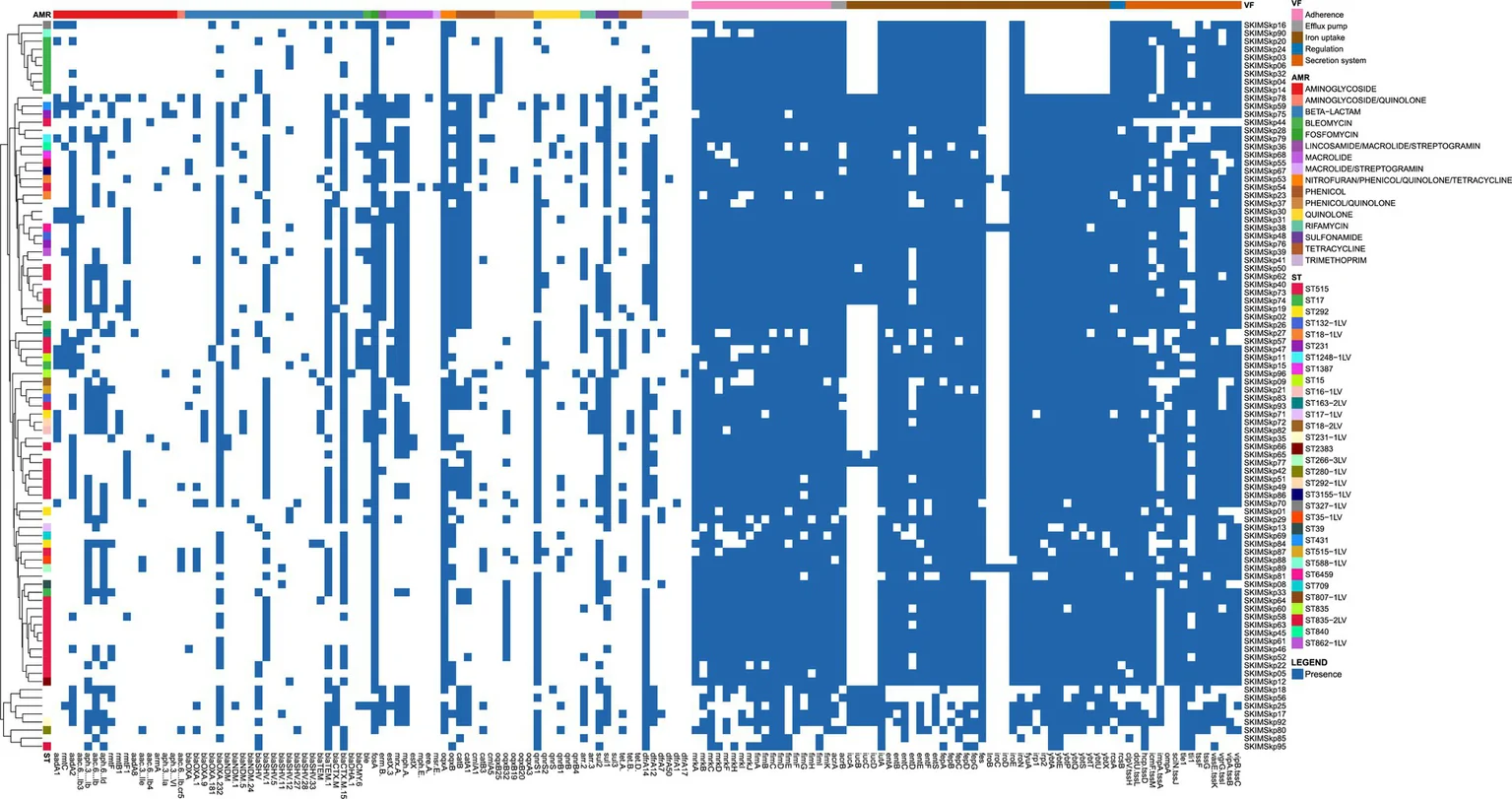
Heatmap depicting antimicrobial resistance (AMR) genes, multilocus sequence types (MLST), and virulence factors (VF) among *K. pneumoniae* study isolates. Rows represent individual isolates, clustered based on gene presence-absence profiles, while columns represent AMR genes (left panel) and VF genes (right panel). Blue colored blocks are indicative of gene presence and absence with no color. The accompanying annotation bars denote VF, AMR classes, and sequence types (STs).

The predominant resistance mechanisms comprised antibiotic inactivation, mediated by genes including *fosA, bla*_CTX-M-15_, *bla*_OXA-232_, *bla*_TEM-1_, and *aadA2* (52–86 isolates); antibiotic target replacement by *dfrA14*, *sul1*, *dfrA12*, and *sul2* (37–64 isolates); antibiotic target protection by *qnrS1* (75 isolates); target modification by *erm(B)* (38 isolates); and efflux-mediated resistance through *oqxA*/*oqxB* (47–85 isolates). The complete distribution of AMR genes is provided in Supplementary Table 3.

A Sankey diagram illustrating the distribution of antibiotic resistance classes across the clinical disease conditions is presented in Supplementary Figure 1.

### 3.5 Carbapenem resistance

Carbapenemase genes were identified in 73 of 90 genomes (81.1%). *bla*_OXA-232_ was the predominant carbapenemase gene (75.6%, 68/90), followed by *bla*_NDM-1_ (14.4%, 13/90), *bla*_NDM-5_ (8.9%, 8/90), *bla*_OXA-181_ (3.3%, 3/90), *bla*_NDM_ (2.2%, 2/90), and *bla*_NDM-24_ (1.1%, 1/90). Among the 27 ST515 isolates, 23 carried *bla*_OXA-232_, including one isolate co-harboring *bla*NDM and another co-harboring *bla*NDM-1 and *bla*_OXA-181_. Five of the ten ST17 isolates carried *bla*_OXA-232_, including one co-harboring *bla*_NDM-1_. ST231 isolates also carried *bla*_OXA-232_ and *bla*_NDM-1_. To further characterize phenotypically carbapenem-resistant isolates without identifiable carbapenemase genes, the distribution of ESBL genes, AmpC β-lactamases, and resistance-associated point mutations (RAPM) is summarized in Figure 4.

**Figure 4:**
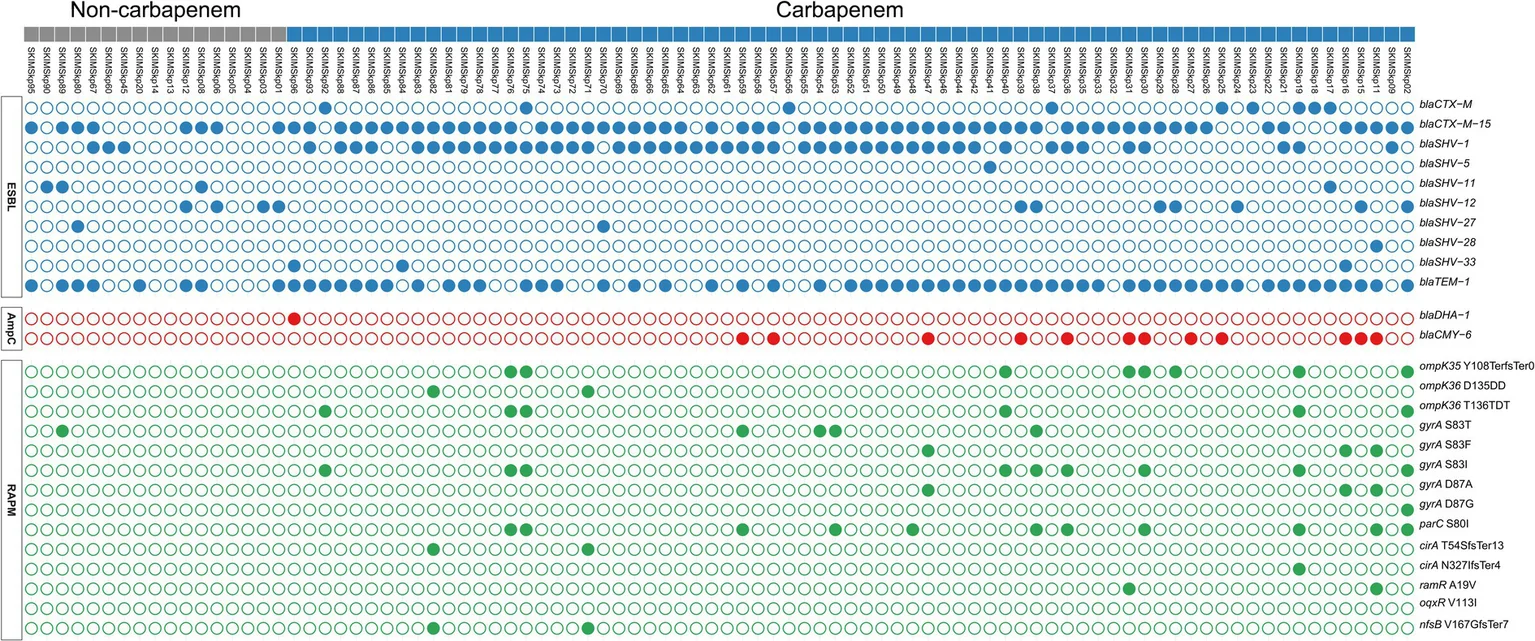
Matrix plot depicting distribution of ESBL, AmpC β-lactamase and resistance-associated point mutations (RAPM) among carbapenemase-positive and carbapenemase-negative *K. pneumoniae* isolates. Filled circles indicate presence of the indicated determinant. The carbapenemase-negative group includes both phenotypically carbapenem-resistant isolates lacking detectable carbapenemase genes and the small number of non-carbapenem-resistant isolates.

### 3.6 Resistance-associated point mutations

Resistance-associated point mutations (RAPM) were identified in ompK35, ompK36, gyrA, parC, cirA, ramR, oqxR, and nfsB. Distribution of Extended spectrum beta-lactamase (ESBL), AmpC genes and RAPM among Carbapenem/Non-carbapenem isolates are depicted in a matrix plot (Figure 4). Among the porin genes, the ompK35_Y108TerfsTer0 frameshift mutation, resulting in a premature stop codon, was detected in eight carbapenem-resistant isolates. In ompK36, the D135DD and T136TDT insertion mutations were identified in two and six carbapenem-resistant isolates, respectively.

Mutations in gyrA were detected at two codons, comprising the substitutions S83T/F/I and D87A/G. The S83T substitution was identified in five isolates, including one non-carbapenem-resistant isolate. The parC S80I mutation was detected in 11 isolates, whereas the ramR A19V mutation was identified in two carbapenem-resistant isolates. The two isolates carrying the oqxR V113I mutation were excluded following re-evaluation of sequencing data.

Two cirA frameshift mutations (cirA_T54SfsTer13 and cirA_N327IfsTer4) introducing premature stop codons were identified. Three isolates carrying cirA mutations also harbored *bla*_NDM-5_ (Supplementary Tables 3, 4). In addition, a frameshift mutation in nfsB, resulting in a premature stop codon, was detected in one isolate. The complete distribution of RAPM is provided in Supplementary Table 4.

### 3.7 Distribution of AMR genes across hospital wards

Figure 5 presents an UpSet-style plot illustrating the distribution of AMR genes across the different hospital wards from which samples were collected. The greatest number of AMR genes, 65 in total, was detected among samples from the OPD and IPD wards, followed by 59 genes among SICU samples. Seventeen AMR genes were shared across all wards: fosA, oqxA, qnrS1, *bla*_OXA-232_, dfrA14, *bla*_TEM-1_, sul1, aadA2, mrx(A), *bla*_SHV-1_, mph(A), catB, catA1, dfrA12, erm(B), aac(6′)-Ib, and arr-2.

**Figure 5:**
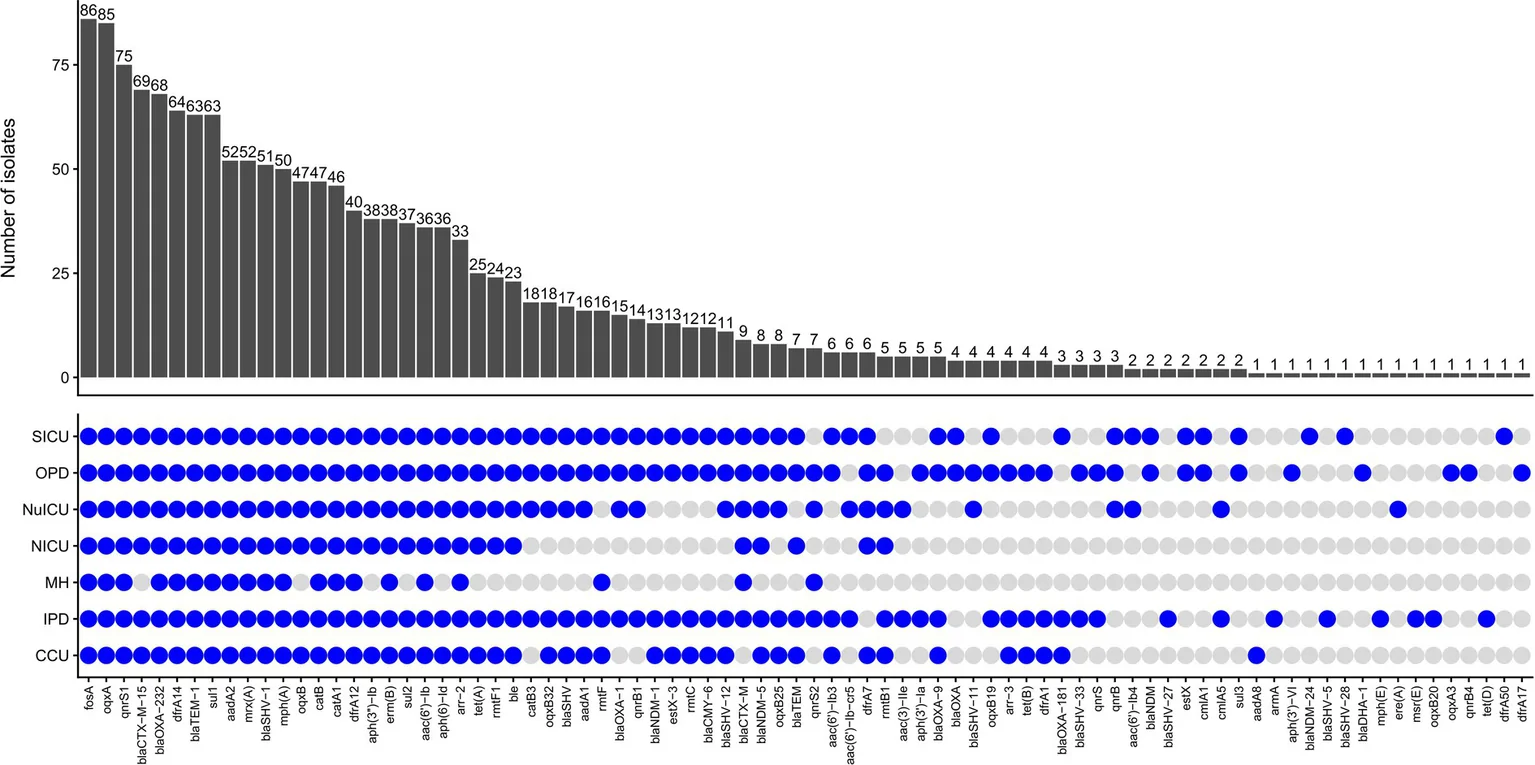
UpSet-style plot visualization of the distribution of antimicrobial resistance genes across hospital wards. The upper bar plot represents the number of *K. pneumoniae* isolates carrying each AMR gene, while the lower matrix indicates the presence (blue circles) or absence (gray circles) of each gene in the seven hospital wards (SICU, IPD, NuICU, CCU, OPD, NICU, and MH). Genes are ordered by decreasing frequency.

### 3.8 Virulence factors

Virulence factors were characterized using the VFDB^4^, and the genes identified were grouped according to their functional roles. The categorization of the isolates considering the presence or absence of virulence factor genes based on MLST can be seen in Figure 3. Virulence factor profiling identified genes involved in adherence (fimABCDEFGHIK), biofilm formation (mrkABCDFHIJ), secretion systems (tssABCDFGHIJKLM, tle1, tli1 and ompA), efflux pumps (acrA/acrB), and regulatory functions (rcsA/rcsB). Iron acquisition systems were well represented and included enterobactin (entABCDEFS, fepABCDG and fes), yersiniabactin (fyuA, irp1, irp2 and ybtAEPQSTUX), aerobactin (iucABCD and iutA), and salmochelin (iroBCDEN). In contrast, the classical hypervirulence-associated regulators rmpA and rmpA2 were not detected among the study isolates.

### 3.9 Plasmid prediction

PlasmidFinder analysis using the local reference database identified a total of 34 plasmid replicons. IncFIB(K)(pCAV1099-114) was the most prevalent replicon, detected in 79/90 (87.8%) genomes, followed by RepB in 72/90 (80.0%), Col440I in 69/90 (76.7%), and ColKP3 in 70/90 (77.8%). Notably, ColKP3 was detected exclusively among carbapenem-resistant isolates. ColpVC, IncC, ColRNAI, IncQ2, and IncFII were likewise detected exclusively among carbapenem-resistant isolates, occurring in 27/90 (30.0%), 13/90 (14.4%), 10/90 (11.1%), 10/90 (11.1%), and 7/90 (7.8%) genomes, respectively. The distribution of plasmid replicons among representative study isolates is depicted in the Sankey plot in Figure 6. The complete distribution of plasmid replicons among the *K. pneumoniae* study isolates is provided in Supplementary Table 5.

**Figure 6:**
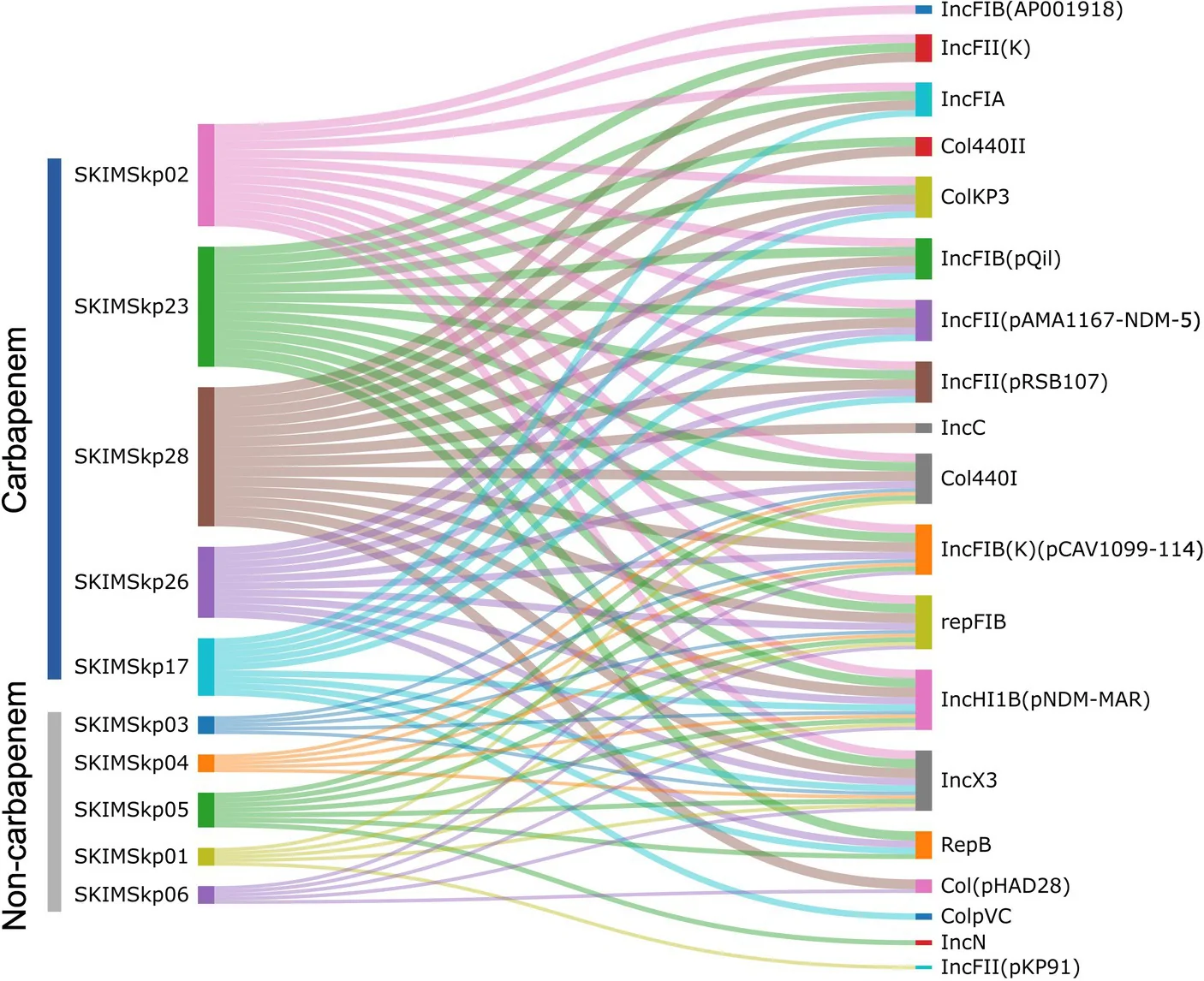
Sankey plot depicting the distribution of plasmid replicons among representative isolates. Representative study isolates are shown on the left and plasmid replicons on the right, with flow width representing the frequency of association.

### 3.10 Phylogenetic analysis

A maximum-likelihood phylogenomic tree based on core genome SNP (cgSNP) alignment was constructed to examine the genetic relationships among the 69 study isolates, the reference strain HS11286, and 63 globally representative *K. pneumoniae* genomes (Figure 7). The phylogeny demonstrated clustering of the study isolates according to their sequence types (STs) and capsular (K) loci. The predominant ST515/KL62 isolates clustered within a clade containing genomes from Asia and North America. Similarly, eight ST17/KL25 isolates clustered with genomes from Asia, Europe, and South America, while ST231/KL51 isolates grouped with genomes originating from Asia, Europe, and Africa. In addition to these major clusters, several study isolates formed distinct phylogenetic branches separate from the predominant lineages.

**Figure 7:**
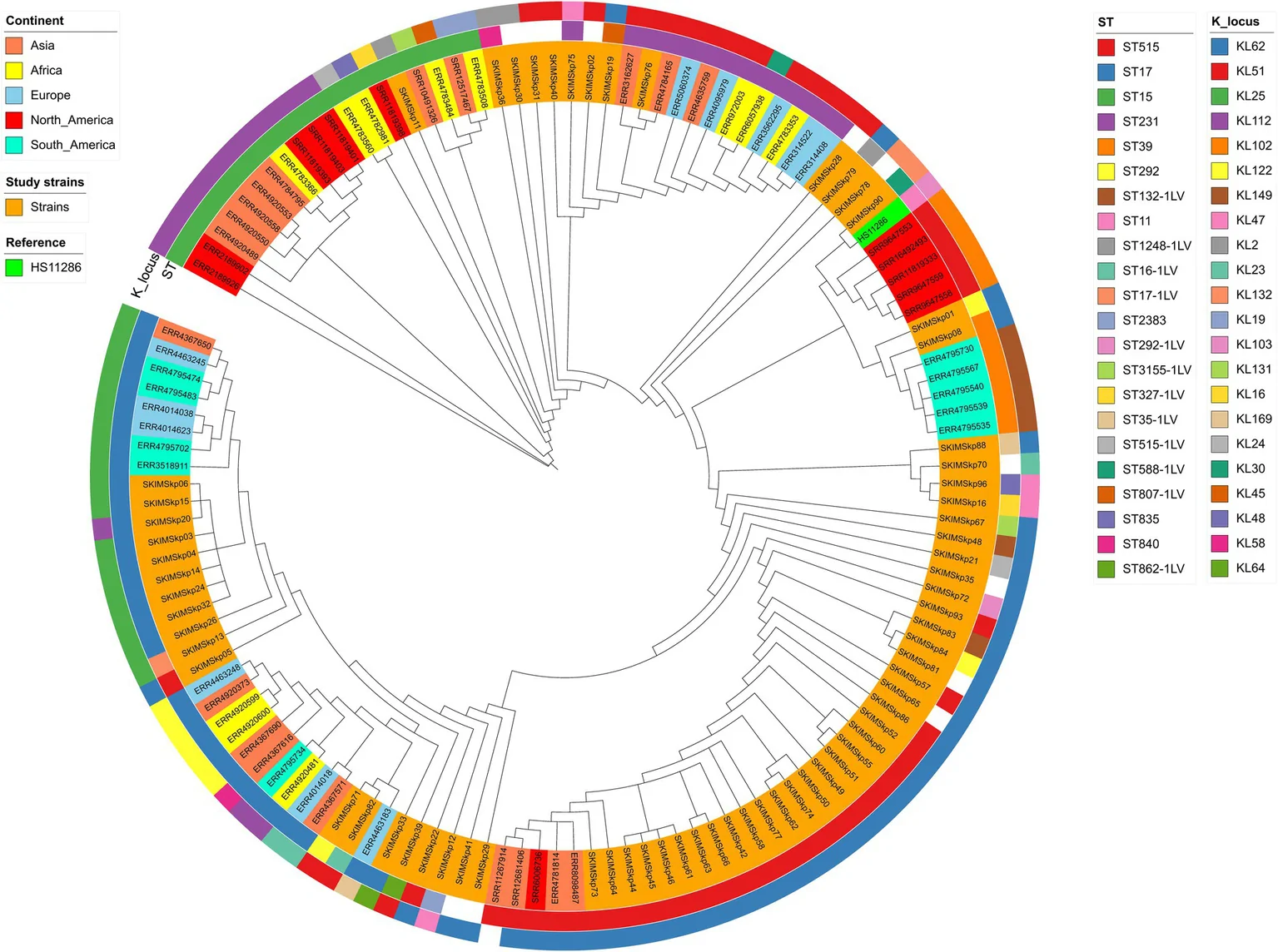
Core genome SNP (cgSNP)-based maximum-likelihood phylogenetic tree of *Klebsiella pneumoniae*. The tree was reconstructed from SNPs identified in the aligned core genome of 69 study isolates, 63 publicly available reference genomes, and the reference strain HS11286. Isolates are color-coded by sequence type (ST). Concentric rings indicate multilocus sequence type (ST) and capsular (K-locus) type.

## 4 Discussion

The present study provides a comprehensive genomic characterization of multidrug-resistant (MDR) carbapenem-resistant *Klebsiella pneumoniae* (CRKP) recovered from a tertiary-care hospital in northern India. Nearly all study isolates (96.9%) exhibited phenotypic resistance to carbapenems, together with high levels of resistance to fluoroquinolones, aminoglycosides, third- and fourth-generation cephalosporins, and β-lactam/β-lactamase inhibitor combinations. This multidrug-resistant phenotype highlights the considerable therapeutic challenges posed by contemporary *K. pneumoniae* in hospital settings, where treatment options are increasingly limited (Navon-Venezia et al., 2017). In contrast, tigecycline resistance remained uncommon, suggesting that it continues to retain activity against the majority of isolates in the present collection, although its clinical use should be guided by antimicrobial stewardship and susceptibility testing.

Whole-genome sequencing further demonstrated a genetically diverse *K. pneumoniae* population, with 32 sequence types identified among 72 isolates assigned complete MLST profiles. Despite this diversity, ST515 emerged as the predominant lineage, followed by ST17, indicating the coexistence of dominant lineages with numerous less frequent sequence types. Similar genomic heterogeneity has been reported in hospital-associated *K. pneumoniae* populations worldwide, reflecting the remarkable adaptability of this species and its ability to acquire antimicrobial resistance through both clonal dissemination and horizontal gene transfer (Lam et al., 2021; Hussain et al., 2023). Interestingly, ST515 has been reported less frequently than internationally recognized high-risk clones such as ST14, ST15, ST147, ST231, and ST258 (Navon-Venezia et al., 2017; Hussain et al., 2023), suggesting that this lineage may have acquired local epidemiological importance within the study setting. However, broader multicentric genomic surveillance will be required to determine whether ST515 represents a regionally prevalent lineage or a localized hospital-associated clone.

Capsular typing revealed KL62 as the predominant K-locus, followed by KL51 and KL25. Capsular polysaccharides are major virulence determinants that facilitate immune evasion, resistance to phagocytosis and complement-mediated killing, biofilm formation, and persistence within both host and environmental niches (Lam et al., 2021; Krūmiņa et al., 2026). Although KL62 has been reported among clinical *K. pneumoniae* isolates from Bangladesh and other parts of South Asia, its epidemiological significance remains less well characterized than capsular types associated with globally disseminated high-risk clones (Hussain et al., 2023). ST515/KL62 was the predominant lineage identified among the sequenced isolates. However, as this study represents a single-center cohort, the prevalence and geographical distribution of this lineage should be interpreted with caution. Larger multicenter genomic surveillance studies will be required to determine its epidemiological significance within India.

Pangenome analysis revealed 13,720 gene families across the 69 genomes studied, comprising 1,574 core genes and 12,146 accessory genes (soft-core, shell and cloud genes). The predominance of accessory genes, particularly the large cloud genome component, highlights the highly dynamic genomic architecture of *K. pneumoniae*. Such an open pangenome is characteristic of this species and reflects continual gene acquisition and loss through horizontal gene transfer, enabling adaptation to diverse ecological niches and antimicrobial selection pressures (Holt et al., 2015; Wyres et al., 2020). The extensive accessory genome observed in the present study is consistent with previous comparative genomic analyses of clinical *K. pneumoniae*, which have demonstrated that mobile genetic elements, including plasmids, transposons, integrative conjugative elements, and prophages, play a central role in shaping genome evolution, antimicrobial resistance, and virulence (Martin and Bachman, 2018; Wyres et al., 2020). Collectively, these findings provide a genomic framework for understanding the remarkable diversity of resistance and virulence determinants observed among the study isolates.

The resistome analysis identified 82 antimicrobial resistance genes spanning 16 antibiotic classes, reflecting the extensive multidrug-resistant phenotype observed among the study isolates. The fosfomycin resistance gene *fosA* was the most prevalent determinant (95.6%), followed by *oqxA* (94.4%), *qnrS1* (83.3%), and the extended-spectrum β-lactamase gene *bla*_CTX-M-15_ (76.7%). The high prevalence of *bla*_CTX-M-15_ is consistent with its widespread global dissemination and its established role as the predominant ESBL determinant among clinical *K. pneumoniae* and other Enterobacterales, contributing substantially to resistance against third-generation cephalosporins (Cantón et al., 2012). Likewise, the widespread occurrence of *qnrS1* together with the OqxAB efflux pump highlights the important contribution of quinolone resistance determinants to the multidrug-resistant phenotype observed in the present study (Jacoby et al., 2014; Li et al., 2015).

Carbapenemase genes were identified in 81.1% (73/90) of the sequenced genomes, with *bla*_OXA-232_ detected in 75.6% (68/90) of isolates, making it the predominant carbapenemase determinant. In comparison, *bla*_NDM-1_ and *bla*_NDM-5_ were detected in 14.4 and 8.9% of isolates, respectively. Occasional co-occurrence of *bla*_OXA-232_ with *bla*_NDM_ variants was also observed, a combination that has previously been reported in clinical *K. pneumoniae* isolates and is associated with enhanced carbapenem resistance (Doi et al., 2014). OXA-232 differs from OXA-48 by five amino-acid substitutions (T104A, N110D, E168Q, S171A, and R214S) (Das et al., 2024). The predominance of *bla*_OXA-232_ observed in the present study is consistent with reports from India and Southeast Asia, where OXA-232-producing *K. pneumoniae* has emerged as an important carbapenem-resistant determinant associated with multidrug resistance and successful clinical dissemination (Shankar et al., 2019; David et al., 2022). Notably, 85.2% (23/27) of the ST515 isolates harbored *bla*_OXA-232_, whereas *bla*_NDM_ variants were distributed across multiple sequence types, indicating that OXA-232 was the predominant carbapenem resistance determinant among the sequenced isolates, with concurrent circulation of NDM-producing lineages. Interestingly, approximately 17 of the 90 genomes (18.9%) lacked an identifiable carbapenemase gene. Most of these isolates nevertheless harbored ESBL determinants, predominantly *bla*_CTX-M-15_, together with alterations in *ompK35* and *ompK36* and other chromosomal resistance-associated mutations. Although ESBL production alone is insufficient to confer carbapenem resistance, its combination with reduced outer membrane permeability resulting from porin alterations has been shown to increase carbapenem resistance in non-carbapenemase-producing *K. pneumoniae* (Tsai et al., 2011; Hamzaoui et al., 2018). Accordingly, carbapenem resistance in this subset of isolates is likely to result from the combined effects of β-lactamase production and impaired membrane permeability rather than acquired carbapenemase production alone.

In addition to acquired resistance genes, several chromosomal mutations associated with antimicrobial resistance were identified. Alterations in the major outer membrane porins OmpK35 and OmpK36, including premature truncation of OmpK35 and the characteristic D135DD and T136TDT insertions in OmpK36, have been associated with reduced outer membrane permeability and enhanced carbapenem resistance, particularly when occurring in combination with carbapenemase production (Tsai et al., 2011; Shankar et al., 2019; Lam et al., 2021). Likewise, mutations in the quinolone resistance-determining regions of gyrA and parC, together with the high prevalence of qnrS1, highlight the contribution of chromosomal and plasmid-mediated quinolone resistance mechanisms to the multidrug-resistant phenotype (Jacoby et al., 2014). The widespread occurrence of the OqxAB efflux pump further supports the contribution of efflux-mediated resistance to the multidrug-resistant phenotype (Li et al., 2015). Notably, *cirA* frameshift mutations were identified in three *bla*_NDM-5_-positive isolates, warranting further investigation of their potential contribution to the resistance phenotype. Although disruption of CirA has recently been implicated in cefiderocol resistance among NDM-producing *K. pneumoniae*, the clinical significance of these mutations in the present study remains uncertain, as cefiderocol susceptibility was not evaluated (McElheny et al., 2021; Mezcord et al., 2024).

The substantial overlap in antimicrobial resistance genes across different hospital wards, with 17 AMR genes shared among isolates from all clinical settings, indicates a conserved multidrug resistance repertoire within the study population. However, the single-center cross-sectional design precludes inference regarding transmission dynamics or ward-specific dissemination.

Virulence profiling demonstrated that the study isolates retained a broad repertoire of genes involved in adherence, biofilm formation, secretion systems, and iron acquisition, all of which contribute to colonization, persistence, and pathogenesis in *K. pneumoniae* (Martin and Bachman, 2018; Krūmiņa et al., 2026). Similar virulence gene profiles have recently been reported among carbapenem-resistant *K. pneumoniae* clinical isolates from China, highlighting the conservation of these core virulence determinants despite extensive antimicrobial resistance (He et al., 2026). Multiple siderophore systems, including enterobactin, yersiniabactin, aerobactin, and salmochelin, were identified, reflecting the importance of efficient iron acquisition during infection (Russo and Marr, 2019; Lam et al., 2021). In contrast, the absence of the classical hypervirulence regulators *rmpA* and *rmpA2* suggests that the study isolates predominantly represent multidrug-resistant rather than hypervirulent lineages, although the coexistence of multiple virulence determinants with antimicrobial resistance remains a significant clinical and public health concern (Russo and Marr, 2019; Lam et al., 2021).

Plasmid analysis demonstrated a high prevalence of IncFIB(K), RepB, Col440I, and ColKP3 replicons, underscoring the important role of plasmids in the acquisition and maintenance of antimicrobial resistance determinants in *K. pneumoniae*. Notably, ColKP3 was detected exclusively among carbapenem-resistant isolates and showed a strong association with *bla*_OXA-232_, consistent with previous reports identifying ColKP3 as an important plasmid associated with *bla*_OXA-232_, particularly among clinical *K. pneumoniae* isolates from India and other parts of Asia (Shankar et al., 2019; David et al., 2022). Furthermore, the exclusive detection of IncC, IncFII, IncQ2, ColRNAI, and ColpVC replicons in carbapenem-resistant isolates supports the contribution of mobile genetic elements to the acquisition and persistence of multidrug resistance (Carattoli, 2009; Carattoli et al., 2014).

Phylogenomic analysis demonstrated that the study isolates clustered primarily according to their sequence types and capsular loci, indicating good concordance between phylogenetic relationships and genomic lineage assignment. The predominant ST515/KL62, ST17/KL25, and ST231/KL51 lineages clustered with publicly available genomes representing the same sequence types from multiple continents, consistent with their placement within internationally distributed clonal lineages. In contrast, several isolates formed distinct phylogenetic branches, reflecting the genomic diversity present within the study collection. These findings highlight the utility of whole-genome sequencing for high-resolution characterization of the population structure and evolutionary relationships of multidrug-resistant *K. pneumoniae* (Holt et al., 2015; Wyres et al., 2020).

A limitation of this study is that MLST and pangenome analyses were performed on subsets of the retained genomes because complete housekeeping-gene profiles or predefined assembly-quality criteria were not met. The study was also limited by its single-centre design, which may restrict the generalizability of the findings. In addition, functional validation of the identified resistance and virulence determinants was not performed. Phenotypic susceptibility testing against newer anti-CPE agents, particularly cefiderocol and aztreonam-avibactam, could not be performed because these agents were not available for reference broth microdilution testing during the study period.

## 5 Conclusion

In conclusion, whole-genome sequencing demonstrated substantial genomic diversity among multidrug-resistant, carbapenem-resistant *Klebsiella pneumoniae* isolates, with an extensive accessory genome, a broad resistome, and predominance of the ST515/KL62 lineage among the sequenced isolates. Larger multicenter genomic studies are needed to determine the prevalence and epidemiological significance of this lineage in India. The high prevalence of *bla*_OXA-232_ and *bla*_CTX-M-15_, representing the predominant carbapenemase and ESBL determinants, respectively, underscores the substantial and multifaceted β-lactam resistance burden in MDR *K. pneumoniae*. Although the isolates carried multiple virulence-associated genes involved in adherence, biofilm formation, secretion systems, and iron acquisition, the absence of the classical hypervirulence regulators *rmpA* and *rmpA2* suggests that the circulating strains predominantly represent multidrug-resistant rather than hypervirulent lineages. Collectively, these findings provide important insights into the genomic epidemiology of carbapenem-resistant *K. pneumoniae* in northern India and underscore the value of whole-genome sequencing for surveillance, monitoring the emergence of high-risk lineages, and informing infection prevention and antimicrobial stewardship strategies.

## Data Availability

The datasets presented in this study can be found in online repositories. The names of the repository/repositories and accession number(s) can be found in the article/Supplementary material.

## Footnotes

1 https://achtman-lab.github.io/GrapeTree/MSTree_holder.html, last accessed 07-07-2026.

2 https://cge.food.dtu.dk/services/PlasmidFinder/, last accessed 25-06-2026

3 https://sankeymatic.com/, last accessed 06-07-2026.

4 https://www.mgc.ac.cn/cgi-bin/VFs/v5/main.cgi, last accessed 14-06-2026.

## Supplementary material

SUPPLEMENTARY FIGURE S1 Sankey plot depicting associations between clinical disease conditions and classes of AMR genes. Disease conditions are shown on the left and AMR gene classes on the right, with flow width representing the frequency of association.
