# Supplementary material for: Comprehensive genomic characterization of multidrug-resistant Klebsiella pneumoniae from North India
Source: Front Microbiol. 2026 Sep 24;17:1831365. doi: 10.3389/fmicb.2026.1831365 (PMC13646146; doi:10.3389/fmicb.2026.1831365)
Supplement: Supplementary file 1 [file Table_1.DOCX]

| **Supplementary Table 1**. Assembly quality metrics of 95 *K. pneumoniae* isolates, together with the corresponding K-locus, specimen source, and antimicrobial resistance gene profiles. Five genomes (shown in grey) lacking the *bla*_SHV_ or harbouring *bla*_ACT_ prompted additional quality assessment & were excluded from the final genomic analyses following re-evaluation of sequencing data, which identified substantial contamination. For pangenome analysis, a further quality filter was applied, and 69 genomes meeting the predefined criteria of ≥90% completeness and <7% contamination were included; the remaining 21 genomes were excluded from pangenome analysis.  **.** | | | | | | | | | | | | |
| --- | --- | --- | --- | --- | --- | --- | --- | --- | --- | --- | --- | --- |
| **Pangenome analysis status** | **Isolate** | **Completeness** | **Contamination** | **Contigs** | **Largest contig** | **Total length** | **GC (%)** | **N50** | **L50** | **K_locus** | **Specimen source** | **Resistant Genes** |
| Included | SKIMSkp01 | 94.39 | 4.04 | 1428 | 56282 | 5682718 | 56.65 | 7066 | 178 | KL62 | Swab | fosA, blaSHV-12, blaTEM-1, blaCTX-M-15, oqxA, oqxB, qnrS1, tet(A), sul1, aph(3'')-Ib, aph(6)-Id, dfrA7 |
| Included | SKIMSkp02 | 99.98 | 1.57 | 266 | 386255 | 5971822 | 56.39 | 94247 | 17 | KL51 | Pus | fosA, blaOXA-232, blaSHV-12, blaTEM-1, blaCTX-M-15, oqxA, oqxB, mrx(A), mph(A), sul1, aadA2, aac(6')-Ib, dfrA12, erm(B), catB, catA1, arr-2, rmtF1, tet(B) |
| Included | SKIMSkp03 | 100 | 0.18 | 85 | 486564 | 5396813 | 57.3 | 276102 | 8 | KL25 | Pleural fluid | fosA, blaSHV-12, oqxA, oqxB25 |
| Included | SKIMSkp04 | 100 | 0.18 | 126 | 285316 | 5409151 | 57.28 | 151999 | 14 | KL25 | CSF | fosA, blaSHV, oqxA, dfrA14, oqxB25 |
| Included | SKIMSkp05 | 99.97 | 6.8 | 938 | 234532 | 5741430 | 56.7 | 17717 | 63 | KL62 | Pus | fosA, blaSHV, oqxA, oqxB, qnrS1, dfrA14 |
| Included | SKIMSkp06 | 100 | 0.13 | 130 | 287737 | 5401884 | 57.26 | 114763 | 17 | KL25 | ETA | fosA, blaSHV-12, blaCTX-M-15, oqxA, oqxB25 |
| Excluded from both AMR genomic and pangenome analyses-contamination | SKIMSkp07 | 78.49 | 16.96 | 4489 | 39411 | 6552958 | 55.12 | 1814 | 760 | KL62 | Pus | fosA, sul2, blaOXA-232, blaTEM-1, blaCTX-M-15, qnrS1, mrx(A), tet(A), sul1, aadA2, aph(3'')-Ib, aac(6')-Ib, dfrA12, aph(6)-Id, dfrA7, catA1 |
| Included | SKIMSkp08 | 100 | 0.13 | 86 | 371152 | 5464133 | 57.32 | 132572 | 12 | KL62 | Sputum | fosA, sul2, blaSHV-11, blaTEM-1, blaCTX-M-15, oqxA, qnrS1, sul1, aph(3'')-Ib, aph(6)-Id, dfrA7, oqxB32 |
| Excluded from pangenome - QC threshold | SKIMSkp09 | 98.42 | 9.04 | 2111 | 48731 | 5987174 | 56.16 | 4757 | 297 | KL62 | Pus | fosA, sul2, blaOXA-232, blaSHV-1, blaTEM, blaCTX-M-15, oqxA, oqxB, qnrS1, mrx(A), mph(A), sul1, dfrA14, aph(3'')-Ib, aph(6)-Id, erm(B), dfrA7, catA1, arr-2 |
| Included | SKIMSkp11 | 100 | 0.96 | 135 | 288822 | 5747973 | 56.9 | 116687 | 16 | KL2 | ETA | fosA, blaOXA-181, blaNDM-1, blaSHV-28, blaTEM-1, blaCTX-M-15, blaCMY-6, ble, oqxA, oqxB, qnrS1, estX-3, aadA1, mrx(A), mph(A), rmtC, tet(A), sul1, aadA2, aac(6')-Ib3 |
| Included | SKIMSkp12 | 100 | 6.68 | 547 | 109760 | 6168720 | 56.16 | 20540 | 85 | KL47 | Urine | fosA, sul2, blaSHV-12, blaTEM-1, blaCTX-M-15, oqxA, oqxB, qnrS1, dfrA14, aph(3'')-Ib |
| Included | SKIMSkp13 | 100 | 5.69 | 743 | 145212 | 5883450 | 56.69 | 16764 | 78 | KL25 | Pus | fosA, blaSHV, oqxA, oqxB, sul1, dfrA14, aac(6')-Ib |
| Included | SKIMSkp14 | 100 | 0.12 | 159 | 373876 | 5415940 | 57.24 | 99574 | 16 | KL25 | Drain Fluid | fosA, blaSHV, oqxA, sul1, aadA2, oqxB25, dfrA12 |
| Included | SKIMSkp15 | 100 | 2.16 | 312 | 175574 | 5963597 | 56.62 | 45974 | 35 | KL25 | Urine | fosA, blaOXA-232, blaNDM-1, blaSHV-12, blaTEM-1, blaCTX-M-15, blaCMY-6, ble, oqxA, qnrS1, estX-3, aadA1, mrx(A), mph(A), rmtC, tet(A), sul1, aadA2, aac(6')-Ib3, oqxB25, qnrS2 |
| Included | SKIMSkp16 | 99.99 | 3.42 | 315 | 229672 | 6133302 | 56.19 | 51382 | 36 | KL47 | BAL | fosA, blaOXA-181, blaNDM-1, blaSHV-33, blaTEM-1, blaCTX-M-15, blaCMY-6, ble, oqxA, oqxB, qnrS1, estX-3, aadA1, mrx(A), mph(A), rmtC, tet(A), sul1, aadA2 |
| Excluded from pangenome - QC threshold | SKIMSkp17 | 97.91 | 12.01 | 2926 | 34119 | 6317181 | 55.69 | 3059 | 554 | KL62 | ETA | fosA, sul2, blaOXA-232, blaSHV-11, blaTEM-1, blaCTX-M, oqxA, oqxB, qnrS1, mrx(A), mph(A), tet(A), sul1, aadA2, dfrA14, aph(3'')-Ib, aac(6')-Ib, dfrA12, aph(6)-Id, erm(B), catB, dfrA7, catA1, arr-2 |
| Excluded from pangenome - QC threshold | SKIMSkp18 | 77.31 | 9.97 | 3461 | 48731 | 4336485 | 54.61 | 1350 | 695 | KL62 | Pus | fosA, sul2, blaOXA-232, blaSHV, blaTEM-1, blaCTX-M, oqxA, mrx(A), mph(A), sul1, aadA2, dfrA14, aph(3'')-Ib, aac(6')-Ib, dfrA12, aph(6)-Id, erm(B), catB, catA1, arr-2, qnrS, rmtF |
| Included | SKIMSkp19 | 100 | 1.26 | 398 | 142161 | 5980369 | 56.51 | 39807 | 50 | KL62 | Swab | fosA, sul2, blaOXA-232, blaNDM-5, blaSHV-1, blaTEM-1, blaCTX-M, ble, oqxA, oqxB, qnrS1, mrx(A), mph(A), tet(A), sul1, aadA2, aph(3'')-Ib, aac(6')-Ib, dfrA12, aph(6)-Id, erm(B), catB, catA1, arr-2, rmtB1, rmtF1 |
| Included | SKIMSkp20 | 100 | 1.34 | 354 | 152919 | 5766397 | 56.61 | 39412 | 44 | KL112 | Swab | fosA, blaSHV, blaTEM-1, oqxA, qnrS1, estX-3, aadA1, aadA2, oqxB25, cmlA1, sul3 |
| Included | SKIMSkp21 | 99.89 | 6.47 | 942 | 66272 | 6180652 | 56.17 | 13703 | 124 | KL62 | Sputum | fosA, sul2, blaOXA-1, blaOXA-232, blaSHV-1, blaTEM-1, blaCTX-M-15, oqxA, oqxB, qnrS1, sul1, dfrA14, aph(3'')-Ib, aac(6')-Ib, aph(6)-Id, erm(B), catB, catA1, arr-2, rmtF, catB3 |
| Included | SKIMSkp22 | 100 | 3.99 | 624 | 227517 | 5902685 | 56.57 | 23371 | 62 | KL62 | Sputum | fosA, sul2, blaOXA-232, blaSHV, blaTEM-1, blaCTX-M-15, oqxA, oqxB, qnrS1, dfrA14, aph(3'')-Ib, aph(6)-Id |
| Excluded from pangenome - QC threshold | SKIMSkp23 | 99.95 | 8.56 | 1459 | 72089 | 6516456 | 55.74 | 8446 | 204 | KL51 | Pus | fosA, blaOXA-232, blaSHV, blaCTX-M, oqxA, oqxB, qnrS1, mrx(A), mph(A), sul1, aadA2, aac(6')-Ib, dfrA12, erm(B), catB, catA1, arr-2, rmtF |
| Included | SKIMSkp24 | 100 | 0.88 | 174 | 377036 | 5461860 | 57.05 | 144590 | 13 | KL25 | Pus | fosA, blaOXA-232, blaSHV-12, blaTEM-1, oqxA, dfrA14, oqxB25, qnrS2 |
| Excluded from pangenome - QC threshold | SKIMSkp25 | 96.42 | 8.03 | 2451 | 77884 | 6753728 | 55.39 | 4383 | 376 | KL51 | Pus | fosA, blaOXA-1, blaOXA-232, blaNDM-1, blaSHV, blaTEM-1, blaCTX-M, blaCMY-6, ble, oqxA, estX-3, mrx(A), mph(A), rmtC, sul1, aadA2, dfrA14, aac(6')-Ib, dfrA12, erm(B), catB, catA1, arr-2, catB3, qnrB1 |
| Included | SKIMSkp26 | 100 | 1.03 | 456 | 145903 | 5754151 | 56.85 | 33688 | 51 | KL25 | Drain Fluid | fosA, blaOXA-232, blaSHV, blaTEM-1, blaCTX-M-15, oqxA, oqxB, qnrS1, mrx(A), mph(A), aac(6')-Ib, catB, catA1, arr-2, rmtF |
| Excluded from pangenome - QC threshold | SKIMSkp27 | 93.71 | 9.24 | 2108 | 44949 | 6261593 | 56.03 | 5191 | 300 | KL62 | ETA | fosA, sul2, blaOXA-232, blaNDM-1, blaSHV, blaTEM-1, blaCTX-M-15, blaCMY-6, ble, oqxA, oqxB, qnrS1, estX-3, mrx(A), mph(A), rmtC, tet(A), sul1, aac(6')-Ib3, dfrA14, aph(3'')-Ib, aph(6)-Id, dfrA7, arr-2, rmtF, aadA8 |
| Included | SKIMSkp28 | 99.84 | 2.18 | 443 | 161195 | 6208867 | 55.97 | 34239 | 47 | KL51 | Pus | fosA, sul2, blaOXA-232, blaSHV-12, blaTEM-1, blaCTX-M-15, oqxA, qnrS1, mrx(A), mph(A), sul1, aadA2, aph(3'')-Ib, aph(6)-Id, erm(B), catB, catA1, rmtF1 |
| Included | SKIMSkp29 | 100 | 4.03 | 1019 | 171692 | 6151406 | 56.21 | 15674 | 97 | KL62 | Swab | fosA, sul2, blaNDM-24, blaSHV-12, blaTEM-1, blaCTX-M-15, ble, oqxA, qnrS1, mrx(A), tet(A), sul1, dfrA14, aph(3'')-Ib, aac(6')-Ib, aph(6)-Id, catB, dfrA7, oqxB32 |
| Included | SKIMSkp30 | 99.76 | 1.86 | 390 | 240180 | 6472097 | 55.69 | 49131 | 36 | KL2 | Pus | blaOXA-232, blaNDM-1, blaSHV-1, blaTEM-1, blaCTX-M-15, blaCMY-6, ble, oqxA, oqxB, qnrS1, estX-3, aadA1, mrx(A), mph(A), rmtC, tet(A), aadA2, dfrA12, erm(B), catB, catA1, rmtF1 |
| Included | SKIMSkp31 | 98.66 | 3.96 | 434 | 194378 | 6529015 | 55.68 | 36421 | 50 | KL51 | Sputum | fosA, blaOXA-232, blaNDM-1, blaSHV-1, blaTEM-1, blaCTX-M-15, blaCMY-6, ble, oqxA, oqxB, qnrS1, estX-3, aadA1, mrx(A), mph(A), rmtC, tet(A), sul1, aadA2, aac(6')-Ib3, dfrA14, dfrA12, erm(B), catB, catA1, rmtF1, qnrB1 |
| Included | SKIMSkp32 | 100 | 0.16 | 101 | 468422 | 5425680 | 57.26 | 285343 | 8 | KL25 | BAL | fosA, blaOXA-232, blaSHV, blaCTX-M-15, oqxA, aadA2, oqxB25, dfrA12 |
| Included | SKIMSkp33 | 100 | 1.31 | 527 | 244776 | 6293685 | 56.12 | 38422 | 43 | KL64 | Sputum | fosA, sul2, blaOXA-232, blaSHV, blaTEM-1, blaCTX-M-15, oqxA, qnrS1, mrx(A), mph(A), sul1, dfrA14, aph(3'')-Ib, aac(6')-Ib, aph(6)-Id, catB, catA1, rmtF, oqxB32 |
| Excluded from both pangenome & AMR genomic analyses-contamination | SKIMSkp34 | 100 | 84.27 | 772 | 178570 | 11126254 | 55.61 | 34071 | 95 | KL62 | Drain Fluid | fosA, sul2, blaOXA-1, blaOXA-232, blaSHV-1, blaTEM-1, blaCTX-M-15, blaACT-15, oqxA, oqxB, qnrS1, mrx(A), mph(A), tet(A), sul1, aadA2, dfrA14, aph(3'')-Ib, dfrA12, aph(6)-Id, erm(B), catB, catA1, rmtF1, catB3, qnrB1, aac(6')-Ib-cr5, aac(3)-IIe |
| Included | SKIMSkp35 | 100 | 5.41 | 659 | 119300 | 6052387 | 56.21 | 22836 | 71 | KL62 | Pus | fosA, blaOXA-9, blaOXA-232, blaNDM, blaSHV-1, blaTEM-1, blaCTX-M-15, ble, oqxA, oqxB, qnrS1, aadA1, mrx(A), mph(A), sul1, aadA2, dfrA14, dfrA12, catB, rmtF, aac(6')-Ib4, estX |
| Included | SKIMSkp36 | 92.74 | 6.84 | 1825 | 79675 | 6115313 | 56.11 | 5947 | 236 | KL2 | Urine | fosA, blaOXA-232, blaNDM-1, blaSHV-1, blaTEM-1, blaCTX-M-15, blaCMY-6, ble, oqxA, estX-3, mrx(A), mph(A), rmtC, tet(A), sul1, dfrA14, aac(6')-Ib, dfrA12, erm(B), catB, catA1, arr-2, qnrS |
| Excluded from pangenome - QC threshold | SKIMSkp37 | 95.51 | 7.83 | 1865 | 35881 | 6086399 | 56.32 | 5133 | 300 | KL62 | Swab | fosA, blaOXA-232, blaSHV-1, blaTEM-1, blaCTX-M, oqxA, qnrS1, mrx(A), mph(A), sul1, aadA2, dfrA14, aac(6')-Ib, dfrA12, qnrS2, erm(B), catB, catA1, arr-2, rmtF |
| Excluded from pangenome - QC threshold | SKIMSkp38 | 100 | 86.52 | 882 | 273788 | 11454211 | 57.47 | 38405 | 78 | KL51 | Pleural fluid | fosA, blaOXA-232, blaSHV-12, blaTEM-1, blaCTX-M-15, oqxA, oqxB, qnrS1, mrx(A), mph(A), sul1, aadA2, dfrA12, erm(B), catB, catA1, rmtF1 |
| Included | SKIMSkp39 | 100 | 1.42 | 345 | 472703 | 6626182 | 55.88 | 79798 | 22 | KL51 | Sputum | fosA, blaOXA-232, blaNDM-1, blaSHV-12, blaTEM-1, blaCTX-M-15, blaCMY-6, ble, oqxA, oqxB, qnrS1, mrx(A), mph(A), rmtC, tet(A), sul1, aadA2, aac(6')-Ib, dfrA12, erm(B), catB, catA1, arr-2, rmtF1 |
| Included | SKIMSkp40 | 99.91 | 2.3 | 366 | 227401 | 6026992 | 56.48 | 53933 | 31 | KL51 | Urine | fosA, sul2, blaOXA-232, blaSHV-1, blaTEM-1, blaCTX-M-15, oqxA, oqxB, qnrS1, mrx(A), mph(A), sul1, aadA2, dfrA14, aph(3'')-Ib, dfrA12, qnrS2, aph(6)-Id, erm(B), catB, catA1, rmtF1 |
| Included | SKIMSkp41 | 99.93 | 3.1 | 421 | 250224 | 6497110 | 55.9 | 59990 | 29 | KL62 | Pus | fosA, blaOXA-1, blaOXA-232, blaNDM-5, blaSHV-5, blaTEM-1, blaCTX-M-15, ble, oqxA, oqxB, qnrS1, mrx(A), mph(A), sul1, aadA2, aac(6')-Ib, dfrA12, erm(B), catB, catA1, arr-2, rmtF1, catB3, qnrB1 |
| Included | SKIMSkp42 | 100 | 0.59 | 142 | 513205 | 5520228 | 57.12 | 152203 | 12 | KL62 | Pus | fosA, blaOXA-232, blaSHV-1, blaTEM-1, blaCTX-M-15, oqxA, oqxB, qnrS1, sul1, aadA2, dfrA14, erm(B), catB, catA1, rmtF1 |
| Excluded from both pangenome & AMR genomic analyses-contamination | SKIMSkp43 | 100 | 62.92 | 1400 | 212388 | 9862446 | 55.76 | 13338 | 150 | KL62 | Pus | fosA, blaOXA-232, blaSHV, blaCTX-M-15, blaACT-15, oqxA, oqxB, qnrS1, mrx(A), mph(A), tet(A), sul1, aadA2, dfrA14, dfrA12, erm(B), catB, catA1, rmtF, qnrB1, aac(3)-IIe |
| Included | SKIMSkp44 | 100 | 0.91 | 250 | 394845 | 5730140 | 56.82 | 70740 | 25 | KL62 | Drain Fluid | fosA, blaOXA-232, blaSHV-1, blaTEM-1, blaCTX-M-15, oqxA, oqxB, qnrS1, sul1, dfrA14, erm(B), catB, catA1, arr-2, rmtF1, aac(6')-Ib4 |
| Included | SKIMSkp45 | 100 | 0.54 | 70 | 964627 | 5405793 | 57.3 | 349782 | 6 | KL62 | Sputum | fosA, blaSHV-1, oqxA, qnrS1, dfrA14, oqxB32 |
| Included | SKIMSkp46 | 99.95 | 0.56 | 118 | 1059442 | 5398950 | 57.22 | 172353 | 9 | KL62 | Pus | fosA, blaOXA, blaOXA-232, blaSHV-1, blaTEM-1, blaCTX-M-15, oqxA, qnrS1, sul1, aadA2, dfrA14, aac(6')-Ib, rmtF, oqxB32 |
| Excluded from pangenome - QC threshold | SKIMSkp47 | 99.99 | 8.02 | 1771 | 113957 | 6037353 | 56.3 | 6703 | 207 | KL2 | ETA | fosA, blaOXA-181, blaNDM-1, blaSHV-1, blaTEM-1, blaCTX-M-15, blaCMY-6, ble, oqxA, qnrS1, estX-3, aadA1, mrx(A), mph(A), rmtC, tet(A), sul1, aadA2, dfrA14, aac(6')-Ib, arr-2 |
| Included | SKIMSkp48 | 100 | 2.57 | 307 | 291869 | 6246818 | 56.31 | 88131 | 22 | KL62 | Pus | fosA, blaOXA-232, blaSHV-1, blaTEM-1, blaCTX-M-15, oqxA, oqxB, qnrS1, mrx(A), mph(A), sul1, aadA2, dfrA12, erm(B), catB, catA1, rmtF1 |
| Included | SKIMSkp49 | 100 | 0.81 | 271 | 213594 | 5763691 | 56.91 | 78351 | 22 | KL62 | Pus | fosA, sul2, blaOXA-1, blaOXA-232, blaSHV-1, blaTEM-1, blaCTX-M-15, oqxA, oqxB, qnrS1, mrx(A), mph(A), tet(A), sul1, aadA2, dfrA14, aph(3'')-Ib, dfrA12, aph(6)-Id, catB, catA1, rmtF1, catB3, qnrB1, aac(6')-Ib-cr5 |
| Included | SKIMSkp50 | 100 | 1.42 | 423 | 182669 | 5828608 | 56.81 | 35778 | 44 | KL62 | Swab | fosA, sul2, blaOXA-232, blaSHV-1, blaTEM-1, blaCTX-M-15, oqxA, oqxB, qnrS1, mrx(A), mph(A), aadA2, dfrA14, aph(3'')-Ib, aac(6')-Ib, dfrA12, aph(6)-Id, erm(B), catB, catA1, arr-2 |
| Included | SKIMSkp51 | 100 | 1.69 | 518 | 168849 | 6088407 | 56.38 | 27034 | 61 | KL62 | Pus | fosA, sul2, blaOXA-232, blaSHV-1, blaTEM-1, blaCTX-M-15, oqxA, oqxB, qnrS1, sul1, aadA2, dfrA14, aph(3'')-Ib, dfrA12, catB, catA1, rmtF1, qnrB1 |
| Included | SKIMSkp52 | 100 | 1.52 | 356 | 169460 | 5852861 | 56.77 | 41273 | 42 | KL62 | Sputum | fosA, blaOXA-232, blaSHV-1, blaTEM-1, blaCTX-M-15, oqxA, qnrS1, dfrA14, aac(6')-Ib, catA1, arr-2, rmtF, oqxB32 |
| Excluded from pangenome - QC threshold | SKIMSkp53 | 100 | 76.41 | 733 | 218924 | 11196459 | 57.49 | 47928 | 69 | KL62 | ETA | fosA, blaOXA-232, blaNDM-5, blaSHV-1, blaTEM, blaCTX-M-15, ble, oqxA, qnrS1, mrx(A), mph(A), sul1, aadA2, dfrA14, dfrA12, erm(B), catB, catA1, rmtF1, oqxB19, dfrA50, qnrB |
| Excluded from pangenome - QC threshold | SKIMSkp54 | 100 | 88.64 | 1013 | 258329 | 11229230 | 57.53 | 21445 | 140 | KL62 | Pus | fosA, blaOXA-1, blaOXA-232, blaSHV-1, blaTEM-1, blaCTX-M-15, oqxA, oqxB, qnrS1, sul1, aadA2, dfrA14, aac(6')-Ib, dfrA12, catB, arr-2, catB3, armA, mph(E), msr(E) |
| Included | SKIMSkp55 | 100 | 3.48 | 531 | 125815 | 6125454 | 56.41 | 25627 | 65 | KL62 | Urine | fosA, blaOXA, blaOXA-232, blaSHV-1, blaCTX-M-15, oqxA, oqxB, qnrS1, mrx(A), mph(A), sul1, aadA2, dfrA14, aac(6')-Ib, dfrA12, erm(B), catB, catA1, arr-2, catB3 |
| Excluded from pangenome - QC threshold | SKIMSkp56 | 74.1 | 8.06 | 2835 | 34167 | 3646874 | 54.82 | 1371 | 444 | KL62 | Urine | blaOXA-232, blaSHV, blaCTX-M, qnrS1, mrx(A), mph(A), sul1, aadA2, dfrA14, aac(6')-Ib, dfrA12, erm(B), catB, catA1, arr-2, rmtF |
| Included | SKIMSkp57 | 99.99 | 5.99 | 548 | 143365 | 5938943 | 56.64 | 23564 | 63 | KL62 | Urine | fosA, blaOXA-232, blaSHV-1, blaTEM-1, blaCTX-M-15, blaCMY-6, oqxA, qnrS1, mrx(A), mph(A), rmtC, tet(A), sul1, aadA2, aac(6')-Ib3, dfrA14, arr-2, oqxB32 |
| Included | SKIMSkp58 | 100 | 0.69 | 141 | 391873 | 5540318 | 57.1 | 128566 | 13 | KL62 | Pus | fosA, blaOXA-232, blaSHV-1, blaCTX-M-15, oqxA, qnrS1, sul1, aadA2, dfrA14, rmtF1, oqxB32 |
| Excluded from pangenome - QC threshold | SKIMSkp59 | 100 | 28.29 | 866 | 170721 | 7863800 | 55.3 | 35018 | 58 | KL2 | Urine | fosA, blaOXA-1, blaOXA-232, blaNDM-1, blaSHV-1, blaTEM-1, blaCTX-M-15, blaCMY-6, ble, oqxA, qnrS1, estX-3, aadA1, mrx(A), mph(A), rmtC, tet(A), sul1, aadA2, aac(6')-Ib3, qnrS2, erm(B), catB, catA1, arr-2, rmtF, catB3, qnrB1, oqxB20, tet(D), aph(3')-Ia |
| Included | SKIMSkp60 | 100 | 1 | 379 | 143366 | 5892921 | 56.58 | 34192 | 48 | KL62 | Pus | fosA, blaSHV-1, oqxA, qnrS1, sul1, dfrA14, arr-2, oqxB32 |
| Included | SKIMSkp61 | 100 | 0.53 | 71 | 791456 | 5415982 | 57.26 | 349751 | 6 | KL62 | Pus | fosA, blaOXA-232, blaSHV-1, oqxA, qnrS1, dfrA14, oqxB32 |
| Included | SKIMSkp62 | 100 | 3.34 | 609 | 161670 | 6163841 | 56.29 | 25109 | 68 | KL62 | Urine | fosA, sul2, blaOXA-232, blaSHV-1, blaTEM-1, blaCTX-M-15, oqxA, oqxB, qnrS1, mrx(A), mph(A), sul1, aadA2, dfrA14, aph(3'')-Ib, aac(6')-Ib, dfrA12, qnrS2, aph(6)-Id, erm(B), catB, catA1, arr-2 |
| Included | SKIMSkp63 | 100 | 0.62 | 188 | 367276 | 5527346 | 57.18 | 83262 | 21 | KL62 | Swab | fosA, blaOXA-232, blaSHV-1, oqxA, qnrS1, dfrA14, oqxB32 |
| Included | SKIMSkp64 | 100 | 0.66 | 220 | 245388 | 5599770 | 57.07 | 80527 | 23 | KL62 | Drain Fluid | fosA, sul2, blaOXA-232, blaSHV-1, blaTEM, blaCTX-M-15, oqxA, qnrS1, dfrA14, aph(3'')-Ib, aph(6)-Id, catB, catA1, oqxB32 |
| Included | SKIMSkp65 | 99.54 | 3.68 | 309 | 285992 | 5748946 | 56.92 | 72644 | 22 | KL62 | Pus | fosA, blaOXA-232, blaSHV-1, blaTEM-1, blaCTX-M-15, qnrS1, mrx(A), mph(A), sul1, aadA2, dfrA14, dfrA12, catB, catA1, rmtF1 |
| Included | SKIMSkp66 | 100 | 0.57 | 132 | 341269 | 5465349 | 57.23 | 134799 | 15 | KL62 | Urine | fosA, blaOXA-232, blaNDM, blaSHV-1, blaCTX-M-15, oqxA, oqxB, qnrS1, mrx(A), mph(A), tet(A), sul1, aadA2, dfrA14, estX, aph(3')-Ia |
| Included | SKIMSkp67 | 100 | 3.04 | 839 | 78778 | 6218508 | 56.08 | 15288 | 112 | KL62 | Urine | fosA, blaOXA, blaSHV-1, blaTEM-1, blaCTX-M-15, oqxA, mrx(A), mph(A), tet(A), sul1, aadA2, dfrA14, aac(6')-Ib, dfrA12, erm(B), catB, catA1, arr-2, catB3, oqxB19, aph(3')-Ia |
| Excluded from pangenome - QC threshold | SKIMSkp68 | 92.6 | 13.29 | 1743 | 39309 | 5886913 | 56.56 | 5403 | 301 | KL62 | Swab | fosA, blaOXA-232, blaNDM-5, blaSHV-1, blaTEM-1, blaCTX-M-15, ble, oqxA, mrx(A), mph(A), tet(A), sul1, aadA2, dfrA14, aac(6')-Ib, dfrA12, catB, catA1, arr-2, qnrS, rmtF, qnrB |
| Excluded from pangenome - QC threshold | SKIMSkp69 | 97.16 | 7.31 | 1213 | 86084 | 5822872 | 56.58 | 10128 | 134 | KL62 | Sputum | fosA, blaOXA-232, blaSHV-1, blaCTX-M-15, oqxA, oqxB, qnrS1, dfrA14, qnrB1 |
| Included | SKIMSkp70 | 99.98 | 5.41 | 583 | 103453 | 6460292 | 55.97 | 22256 | 84 | KL23 | Sputum | fosA, sul2, blaOXA-1, blaOXA-9, blaOXA-232, blaNDM-1, blaSHV-27, blaTEM-1, blaCTX-M-15, ble, oqxA, qnrS1, aadA1, tet(A), sul1, dfrA14, aph(3'')-Ib, aph(6)-Id, arr-2, catB3, qnrB1, aac(3)-IIe, oqxB19, dfrA1 |
| Included | SKIMSkp71 | 99.98 | 3.34 | 870 | 88287 | 5712061 | 56.93 | 13333 | 103 | KL51 | Swab | fosA, sul2, blaOXA-9, blaOXA-232, blaNDM-5, blaSHV-1, blaTEM, blaCTX-M-15, ble, oqxA, qnrS1, aadA1, mrx(A), mph(A), sul1, dfrA14, aph(3'')-Ib, aac(6')-Ib, aph(6)-Id, rmtB1, oqxB32, tet(B), dfrA1, arr-3 |
| Included | SKIMSkp72 | 100 | 4.06 | 811 | 228921 | 5978435 | 56.59 | 16178 | 85 | KL62 | Drain Fluid | fosA, sul2, blaOXA-9, blaOXA-232, blaNDM-5, blaSHV-1, blaTEM, blaCTX-M-15, ble, oqxA, qnrS1, aadA1, mrx(A), mph(A), sul1, dfrA14, aph(3'')-Ib, aac(6')-Ib, aph(6)-Id, erm(B), rmtB1, oqxB32, tet(B), dfrA1, arr-3 |
| Included | SKIMSkp73 | 100 | 2.09 | 291 | 265836 | 6001622 | 56.71 | 83975 | 21 | KL62 | ETA | fosA, sul2, blaOXA-232, blaSHV-1, blaTEM-1, blaCTX-M-15, oqxA, oqxB, qnrS1, mrx(A), mph(A), sul1, aadA2, dfrA14, aph(3'')-Ib, dfrA12, aph(6)-Id, erm(B), catB, catA1, rmtF1 |
| Included | SKIMSkp74 | 100 | 3.08 | 332 | 302351 | 6293694 | 56.32 | 74030 | 23 | KL62 | Pus | fosA, sul2, blaOXA-232, blaSHV-1, blaTEM-1, blaCTX-M-15, oqxA, oqxB, qnrS1, mrx(A), mph(A), sul1, aadA2, dfrA14, aph(3'')-Ib, dfrA12, aph(6)-Id, erm(B), catB, catA1, rmtF1 |
| Included | SKIMSkp75 | 100 | 6.8 | 890 | 120663 | 6303861 | 56.07 | 14620 | 114 | KL47 | Pus | fosA, blaOXA-1, blaOXA-232, blaNDM-1, blaSHV-1, blaTEM-1, blaCTX-M, ble, oqxA, oqxB, qnrS1, estX-3, aadA1, mrx(A), mph(A), tet(A), sul1, aadA2, aac(6')-Ib, dfrA12, erm(B), catB, catA1, arr-2, rmtF, catB3, aph(3')-Ia, aph(3')-VI |
| Included | SKIMSkp76 | 100 | 0.79 | 143 | 267877 | 5523740 | 57.14 | 110970 | 16 | KL51 | Urine | fosA, blaOXA-232, blaSHV-1, blaCTX-M-15, oqxA, oqxB, qnrS1, mrx(A), mph(A), sul1, aadA2, dfrA12, erm(B), catB, catA1, rmtF1 |
| Included | SKIMSkp77 | 100 | 0.67 | 191 | 329843 | 5632894 | 57.03 | 91730 | 17 | KL62 | Sputum | fosA, blaOXA-232, blaSHV-1, blaCTX-M-15, oqxA, qnrS1, sul1, aadA2, dfrA14, dfrA12, catA1, rmtF1, oqxB32 |
| Included | SKIMSkp78 | 99.84 | 3.72 | 493 | 206814 | 7073963 | 55.32 | 42999 | 41 | KL132 | ETA | fosA, sul2, blaOXA-1, blaNDM-5, blaSHV-1, blaTEM-1, blaCTX-M-15, ble, oqxA, oqxB, qnrS1, aadA1, mrx(A), mph(A), tet(A), sul1, aadA2, dfrA14, aph(3'')-Ib, dfrA12, aph(6)-Id, erm(B), catB, catA1, arr-2, rmtB1, rmtF1, catB3, qnrB1, aac(6')-Ib-cr5, aac(3)-IIe, cmlA5, ere(A) |
| Included | SKIMSkp79 | 99.99 | 2.78 | 309 | 334604 | 6211186 | 56.28 | 88137 | 21 | KL62 | Pus | fosA, sul2, blaOXA-1, blaOXA-232, blaSHV-1, blaTEM-1, blaCTX-M-15, oqxA, oqxB, qnrS1, aadA1, mrx(A), mph(A), sul1, aadA2, aph(3'')-Ib, aac(6')-Ib, dfrA12, aph(6)-Id, erm(B), catB, catA1, rmtF1, catB3, qnrB1, cmlA5 |
| Excluded from pangenome - QC threshold | SKIMSkp80 | 89.57 | 12.06 | 3164 | 55082 | 4971452 | 56.24 | 1891 | 594 | KL62 | Pus | fosA, sul2, blaOXA-1, blaSHV-27, blaTEM-1, blaCTX-M-15, oqxA, qnrS1, tet(A), dfrA14, aph(3'')-Ib, aph(6)-Id, catB3, qnrB1, aac(6')-Ib-cr5, aac(3)-IIe |
| Included | SKIMSkp81 | 99.92 | 5.63 | 1265 | 64955 | 5910271 | 56.35 | 8156 | 189 | KL62 | ETA | fosA, sul2, blaOXA-232, blaSHV-1, blaTEM-1, blaCTX-M-15, oqxA, oqxB, qnrS1, dfrA14, aph(3'')-Ib, aph(6)-Id |
| Included | SKIMSkp82 | 100 | 2.53 | 502 | 245727 | 5956919 | 56.5 | 31786 | 45 | KL51 | Pleural fluid | fosA, sul2, blaOXA-9, blaOXA-232, blaNDM-5, blaSHV-1, blaTEM, blaCTX-M-15, ble, oqxA, qnrS1, aadA1, mrx(A), mph(A), sul1, dfrA14, aph(3'')-Ib, aac(6')-Ib, aph(6)-Id, erm(B), rmtB1, oqxB32, tet(B), dfrA1, arr-3 |
| Included | SKIMSkp83 | 100 | 1.15 | 309 | 289958 | 6152689 | 56.29 | 64089 | 28 | KL62 | BAL | fosA, sul2, blaOXA-232, blaSHV-1, blaTEM-1, blaCTX-M-15, oqxA, oqxB, qnrS1, mrx(A), sul1, aadA2, dfrA14, aph(3'')-Ib, aac(6')-Ib, dfrA12, aph(6)-Id, erm(B), catB, catA1 |
| Included | SKIMSkp84 | 99.32 | 3.25 | 1285 | 39023 | 5349773 | 57.55 | 6136 | 258 | KL62 | Pus | fosA, sul2, blaOXA-232, blaSHV-33, blaTEM, blaCTX-M-15, oqxA, oqxB, qnrS1, dfrA14, aph(3'')-Ib, aac(6')-Ib, aph(6)-Id, rmtF |
| Excluded from pangenome - QC threshold | SKIMSkp85 | 72.11 | 9.34 | 3178 | 48731 | 3743274 | 55.45 | 1202 | 654 | KL62 | Pus | blaOXA-232, blaSHV, blaTEM-1, blaCTX-M-15, qnrS1, dfrA14, aac(6')-Ib, catB, arr-2 |
| Included | SKIMSkp86 | 100 | 0.9 | 301 | 211818 | 5756959 | 56.72 | 73059 | 24 | KL62 | Pus | fosA, sul2, blaOXA-232, blaSHV-1, blaTEM-1, blaCTX-M-15, oqxA, qnrS1, mrx(A), mph(A), sul1, aadA2, dfrA14, aph(3'')-Ib, dfrA12, aph(6)-Id, catB, catA1, rmtF1, oqxB32 |
| Excluded from pangenome - QC threshold | SKIMSkp87 | 100 | 21.73 | 2057 | 78909 | 7927983 | 55.88 | 7979 | 240 | KL62 | ETA | fosA, sul2, blaOXA-1, blaOXA-232, blaSHV-1, blaTEM-1, blaCTX-M-15, oqxA, qnrS1, dfrA14, aph(3'')-Ib, qnrS2, aph(6)-Id, catB3, oqxB32, aac(6')-Ib-cr5, qnrB |
| Included | SKIMSkp88 | 99.97 | 5.25 | 348 | 128251 | 5927155 | 56.54 | 36449 | 55 | KL62 | Pleural fluid | fosA, sul2, blaOXA-1, blaOXA-232, blaSHV-1, blaTEM-1, blaCTX-M-15, oqxA, qnrS1, tet(A), dfrA14, aph(3'')-Ib, aph(6)-Id, catB3, qnrB1, aac(6')-Ib-cr5, aac(3)-IIe, oqxB19 |
| Excluded from pangenome - QC threshold | SKIMSkp89 | 100 | 88 | 458 | 268951 | 11057644 | 57.78 | 69841 | 48 | KL132 | Pus | fosA, sul2, blaOXA-1, blaSHV-11, blaTEM-1, blaCTX-M-15, oqxA, oqxB, tet(A), dfrA14, aph(3'')-Ib, aph(6)-Id, catB3, qnrB1, aac(6')-Ib-cr5, aac(3)-IIe |
| Included | SKIMSkp90 | 100 | 0.43 | 117 | 421832 | 5526719 | 57.09 | 106824 | 16 | KL132 | Sputum | fosA, blaSHV-11, oqxA, oqxB |
| Excluded from both pangenome & AMR genomic analyses-contamination | SKIMSkp91 | 62.56 | 6.5 | 2983 | 49275 | 3534446 | 54.78 | 1204 | 552 | KL62 | Pus | sul2, blaTEM-1, blaCTX-M-15, qnrS1, dfrA14, aph(3'')-Ib, aph(6)-Id |
| Excluded from pangenome - QC threshold | SKIMSkp92 | 95.11 | 8.29 | 2382 | 34166 | 5882024 | 56.05 | 3747 | 400 | KL51 | Pus | fosA, sul2, blaOXA-232, blaSHV, blaTEM-1, blaCTX-M, oqxA, qnrS1, mrx(A), mph(A), sul1, aadA2, dfrA14, aph(3'')-Ib, aac(6')-Ib, dfrA12, aph(6)-Id, erm(B), catB, catA1, arr-2, rmtF |
| Included | SKIMSkp93 | 99.97 | 3.42 | 1003 | 75118 | 6050162 | 56.19 | 10867 | 152 | KL62 | Urine | fosA, sul2, blaOXA, blaOXA-232, blaSHV-1, blaTEM-1, blaCTX-M-15, oqxA, oqxB, qnrS1, mrx(A), mph(A), sul1, aadA2, dfrA14, aph(3'')-Ib, aac(6')-Ib, dfrA12, aph(6)-Id, erm(B), catB, catA1, arr-2, catB3, qnrB1 |
| Excluded from both pangenome & AMR genomic analyses-contamination | SKIMSkp94 | 63.08 | 8.15 | 3095 | 50371 | 3627166 | 55.35 | 1194 | 620 | KL62 | Drain Fluid | sul2, blaTEM-1, blaCTX-M-15, qnrS1, dfrA14, aph(3'')-Ib, aph(6)-Id |
| Excluded from pangenome - QC threshold | SKIMSkp95 | 75.52 | 7.82 | 3500 | 49275 | 4543761 | 55.92 | 1420 | 707 | KL62 | Swab | sul2, blaSHV, blaTEM-1, blaCTX-M-15, oqxB, qnrS1, dfrA14, aph(3'')-Ib, aph(6)-Id |
| Included | SKIMSkp96 | 100 | 1.12 | 382 | 150103 | 5844341 | 56.68 | 34657 | 47 | KL47 | Urine | fosA, blaOXA-1, blaOXA-232, blaSHV-33, blaTEM-1, blaCTX-M-15, blaDHA-1, oqxB, qnrS1, estX-3, mrx(A), mph(A), sul1, aadA2, aac(6')-Ib, cmlA1, sul3, catB3, aph(3')-Ia, arr-3, oqxA3, qnrB4, dfrA17 |
