# Supplementary material for: Comprehensive genomic characterization of multidrug-resistant Klebsiella pneumoniae from North India
Source: Front Microbiol. 2026 Sep 24;17:1831365. doi: 10.3389/fmicb.2026.1831365 (PMC13646146; doi:10.3389/fmicb.2026.1831365)
Supplement: Supplementary file 2 [file Table_2.DOCX]

| Supplementary Table 2. List of isolates selected from the Pasteur Institute database | | | | | | |
| --- | --- | --- | --- | --- | --- | --- |
| **Sl. No.** | **Isolate** | **Continent** | **ST** | **K_locus** | **Country** | **Year** |
| 1 | HS11286 | Reference sequence | ST11 | KL103 | China | 2011 |
| 2 | SRR10491326 | Asia | ST15 | KL131 | China | 2018 |
| 3 | SRR12517467 | Asia | ST15 | KL19 | China | 2015 |
| 4 | ERR4920550 | Asia | ST15 | KL112 | Pakistan | 2016 |
| 5 | ERR4920553 | Asia | ST15 | KL112 | Pakistan | 2016 |
| 6 | ERR4920558 | Asia | ST15 | KL112 | Pakistan | 2016 |
| 7 | ERR4784795 | Asia | ST15 | KL112 | India | 2017 |
| 8 | ERR4920489 | Asia | ST15 | KL112 | India | 2016 |
| 9 | ERR4367571 | Asia | ST17 | KL23 | Thailand | 2018 |
| 10 | ERR4367616 | Asia | ST17 | KL58 | Thailand | 2018 |
| 11 | ERR4367650 | Asia | ST17 | KL25 | Thailand | 2018 |
| 12 | ERR4367690 | Asia | ST17 | KL122 | Thailand | 2018 |
| 13 | ERR4920373 | Asia | ST17 | KL122 | Bangladesh | 2016 |
| 14 | ERR3162627 | Asia | ST231 | KL51 | India | 2017 |
| 15 | ERR4635759 | Asia | ST231 | KL51 | India | 2019 |
| 16 | ERR4784165 | Asia | ST231 | KL51 | India | 2014 |
| 17 | ERR4781814 | Asia | ST515 | KL62 | Philippines | 2017 |
| 18 | ERR8098487 | Asia | ST515 | KL62 | Bangladesh | - |
| 19 | SRR11267914 | Asia | ST515 | - | Qatar | 2018 |
| 20 | SRR12681406 | Asia | ST515 | KL62 | India | 2019 |
| 21 | ERR4782981 | Africa | ST15 | KL48 | Nigeria | 2017 |
| 22 | ERR4783366 | Africa | ST15 | KL112 | Nigeria | 2017 |
| 23 | ERR4783484 | Africa | ST15 | KL45 | Nigeria | 2017 |
| 24 | ERR4783508 | Africa | ST15 | KL19 | Nigeria | 2017 |
| 25 | ERR4783560 | Africa | ST15 | KL24 | Nigeria | - |
| 26 | ERR4920481 | Africa | ST17 | KL112 | Ethiopia | 2016 |
| 27 | ERR4920599 | Africa | ST17 | KL122 | Rwanda | 2016 |
| 28 | ERR4920600 | Africa | ST17 | KL122 | Rwanda | 2016 |
| 29 | ERR972003 | Africa | ST231 | KL51 | Malawi | 2007 |
| 30 | ERR4783353 | Africa | ST231 | KL51 | Nigeria | 2017 |
| 31 | ERR6057938 | Africa | ST231 | KL30 | Malawi | 2017 |
| 32 | ERR4014018 | Europe | ST17 | KL23 | Denmark | 2018 |
| 33 | ERR4014038 | Europe | ST17 | KL25 | Denmark | 2018 |
| 34 | ERR4014623 | Europe | ST17 | KL25 | Denmark | 2018 |
| 35 | ERR4463183 | Europe | ST17 | KL169 | Germany | - |
| 36 | ERR4463245 | Europe | ST17 | KL25 | Germany | - |
| 37 | ERR4463248 | Europe | ST17 | KL122 | Germany | - |
| 38 | ERR314408 | Europe | ST231 | KL51 | UK | 2012 |
| 39 | ERR314522 | Europe | ST231 | KL51 | UK | - |
| 40 | ERR3562295 | Europe | ST231 | KL51 | The Netherlands | 2015 |
| 41 | ERR4095979 | Europe | ST231 | KL51 | UK | 2017 |
| 42 | ERR5060374 | Europe | ST231 | KL51 | The Netherlands | - |
| 43 | ERR2189902 | North_America | ST15 | KL112 | USA | - |
| 44 | ERR2189926 | North_America | ST15 | KL112 | USA | - |
| 45 | SRR11819393 | North_America | ST15 | KL112 | USA | 2017 |
| 46 | SRR11819398 | North_America | ST15 | KL16 | USA | 2018 |
| 47 | SRR11819401 | North_America | ST15 | KL112 | USA | 2017 |
| 48 | SRR11819403 | North_America | ST15 | KL112 | USA | 2016 |
| 49 | SRR6006736 | North_America | ST515 | KL62 | USA | 2017 |
| 50 | SRR9647553 | North_America | ST515 | KL102 | USA | 2018 |
| 51 | SRR9647558 | North_America | ST515 | KL102 | USA | 2018 |
| 52 | SRR9647559 | North_America | ST515 | KL102 | USA | 2018 |
| 53 | SRR11819333 | North_America | ST515 | KL102 | USA | 2018 |
| 54 | SRR16492493 | North_America | ST515 | KL102 | USA | 2021 |
| 55 | ERR3518911 | South_America | ST17 | KL25 | Brazil | 2018 |
| 56 | ERR4795474 | South_America | ST17 | KL25 | Colombia | 2015 |
| 57 | ERR4795483 | South_America | ST17 | KL25 | Colombia | 2015 |
| 58 | ERR4795702 | South_America | ST17 | KL25 | Colombia | 2014 |
| 59 | ERR4795734 | South_America | ST17 | KL112 | Colombia | 2016 |
| 60 | ERR4795535 | South_America | ST39 | KL149 | Colombia | 2016 |
| 61 | ERR4795539 | South_America | ST39 | KL149 | Colombia | 2016 |
| 62 | ERR4795540 | South_America | ST39 | KL149 | Colombia | 2016 |
| 63 | ERR4795567 | South_America | ST39 | KL149 | Colombia | 2016 |
| 64 | ERR4795730 | South_America | ST39 | KL149 | Colombia | 2016 |
