# Supplementary material for: Comprehensive genomic characterization of multidrug-resistant Klebsiella pneumoniae from North India
Source: Front Microbiol. 2026 Sep 24;17:1831365. doi: 10.3389/fmicb.2026.1831365 (PMC13646146; doi:10.3389/fmicb.2026.1831365)
Supplement: Supplementary file 3 [file Table_3.DOCX]

| **Supplementary Table 3.** **Distribution of AMR genes in *K. pneumoniae* study isolates (n=95): Five excluded samples are highlighted in grey rows.** | |
| --- | --- |
| **Isolate** | **Gene** |
| SKIMSkp01 | fosA, blaSHV-12, blaTEM-1, blaCTX-M-15, oqxA, oqxB, qnrS1, tet(A), sul1, aph(3'')-Ib, aph(6)-Id, dfrA7 |
| SKIMSkp02 | fosA, blaOXA-232, blaSHV-12, blaTEM-1, blaCTX-M-15, oqxA, oqxB, mrx(A), mph(A), sul1, aadA2, aac(6')-Ib, dfrA12, erm(B), catB, catA1, arr-2, rmtF1, tet(B) |
| SKIMSkp03 | fosA, blaSHV-12, oqxA, oqxB25 |
| SKIMSkp04 | fosA, blaSHV, oqxA, dfrA14, oqxB25 |
| SKIMSkp05 | fosA, blaSHV, oqxA, oqxB, qnrS1, dfrA14 |
| SKIMSkp06 | fosA, blaSHV-12, blaCTX-M-15, oqxA, oqxB25 |
| SKIMSkp07 | fosA, sul2, blaOXA-232, blaTEM-1, blaCTX-M-15, qnrS1, mrx(A), tet(A), sul1, aadA2, aph(3'')-Ib, aac(6')-Ib, dfrA12, aph(6)-Id, dfrA7, catA1 |
| SKIMSkp08 | fosA, sul2, blaSHV-11, blaTEM-1, blaCTX-M-15, oqxA, qnrS1, sul1, aph(3'')-Ib, aph(6)-Id, dfrA7, oqxB32 |
| SKIMSkp09 | fosA, sul2, blaOXA-232, blaSHV-1, blaTEM, blaCTX-M-15, oqxA, oqxB, qnrS1, mrx(A), mph(A), sul1, dfrA14, aph(3'')-Ib, aph(6)-Id, erm(B), dfrA7, catA1, arr-2 |
| SKIMSkp11 | fosA, blaOXA-181, blaNDM-1, blaSHV-28, blaTEM-1, blaCTX-M-15, blaCMY-6, ble, oqxA, oqxB, qnrS1, estX-3, aadA1, mrx(A), mph(A), rmtC, tet(A), sul1, aadA2, aac(6')-Ib3 |
| SKIMSkp12 | fosA, sul2, blaSHV-12, blaTEM-1, blaCTX-M-15, oqxA, oqxB, qnrS1, dfrA14, aph(3'')-Ib |
| SKIMSkp13 | fosA, blaSHV, oqxA, oqxB, sul1, dfrA14, aac(6')-Ib |
| SKIMSkp14 | fosA, blaSHV, oqxA, sul1, aadA2, oqxB25, dfrA12 |
| SKIMSkp15 | fosA, blaOXA-232, blaNDM-1, blaSHV-12, blaTEM-1, blaCTX-M-15, blaCMY-6, ble, oqxA, qnrS1, estX-3, aadA1, mrx(A), mph(A), rmtC, tet(A), sul1, aadA2, aac(6')-Ib3, oqxB25, qnrS2 |
| SKIMSkp16 | fosA, blaOXA-181, blaNDM-1, blaSHV-33, blaTEM-1, blaCTX-M-15, blaCMY-6, ble, oqxA, oqxB, qnrS1, estX-3, aadA1, mrx(A), mph(A), rmtC, tet(A), sul1, aadA2 |
| SKIMSkp17 | fosA, sul2, blaOXA-232, blaSHV-11, blaTEM-1, blaCTX-M, oqxA, oqxB, qnrS1, mrx(A), mph(A), tet(A), sul1, aadA2, dfrA14, aph(3'')-Ib, aac(6')-Ib, dfrA12, aph(6)-Id, erm(B), catB, dfrA7, catA1, arr-2 |
| SKIMSkp18 | fosA, sul2, blaOXA-232, blaSHV, blaTEM-1, blaCTX-M, oqxA, mrx(A), mph(A), sul1, aadA2, dfrA14, aph(3'')-Ib, aac(6')-Ib, dfrA12, aph(6)-Id, erm(B), catB, catA1, arr-2, qnrS, rmtF |
| SKIMSkp19 | fosA, sul2, blaOXA-232, blaNDM-5, blaSHV-1, blaTEM-1, blaCTX-M, ble, oqxA, oqxB, qnrS1, mrx(A), mph(A), tet(A), sul1, aadA2, aph(3'')-Ib, aac(6')-Ib, dfrA12, aph(6)-Id, erm(B), catB, catA1, arr-2, rmtB1, rmtF1 |
| SKIMSkp20 | fosA, blaSHV, blaTEM-1, oqxA, qnrS1, estX-3, aadA1, aadA2, oqxB25, cmlA1, sul3 |
| SKIMSkp21 | fosA, sul2, blaOXA-1, blaOXA-232, blaSHV-1, blaTEM-1, blaCTX-M-15, oqxA, oqxB, qnrS1, sul1, dfrA14, aph(3'')-Ib, aac(6')-Ib, aph(6)-Id, erm(B), catB, catA1, arr-2, rmtF, catB3 |
| SKIMSkp22 | fosA, sul2, blaOXA-232, blaSHV, blaTEM-1, blaCTX-M-15, oqxA, oqxB, qnrS1, dfrA14, aph(3'')-Ib, aph(6)-Id |
| SKIMSkp23 | fosA, blaOXA-232, blaSHV, blaCTX-M, oqxA, oqxB, qnrS1, mrx(A), mph(A), sul1, aadA2, aac(6')-Ib, dfrA12, erm(B), catB, catA1, arr-2, rmtF |
| SKIMSkp24 | fosA, blaOXA-232, blaSHV-12, blaTEM-1, oqxA, dfrA14, oqxB25, qnrS2 |
| SKIMSkp25 | fosA, blaOXA-1, blaOXA-232, blaNDM-1, blaSHV, blaTEM-1, blaCTX-M, blaCMY-6, ble, oqxA, estX-3, mrx(A), mph(A), rmtC, sul1, aadA2, dfrA14, aac(6')-Ib, dfrA12, erm(B), catB, catA1, arr-2, catB3, qnrB1 |
| SKIMSkp26 | fosA, blaOXA-232, blaSHV, blaTEM-1, blaCTX-M-15, oqxA, oqxB, qnrS1, mrx(A), mph(A), aac(6')-Ib, catB, catA1, arr-2, rmtF |
| SKIMSkp27 | fosA, sul2, blaOXA-232, blaNDM-1, blaSHV, blaTEM-1, blaCTX-M-15, blaCMY-6, ble, oqxA, oqxB, qnrS1, estX-3, mrx(A), mph(A), rmtC, tet(A), sul1, aac(6')-Ib3, dfrA14, aph(3'')-Ib, aph(6)-Id, dfrA7, arr-2, rmtF, aadA8 |
| SKIMSkp28 | fosA, sul2, blaOXA-232, blaSHV-12, blaTEM-1, blaCTX-M-15, oqxA, qnrS1, mrx(A), mph(A), sul1, aadA2, aph(3'')-Ib, aph(6)-Id, erm(B), catB, catA1, rmtF1 |
| SKIMSkp29 | fosA, sul2, blaNDM-24, blaSHV-12, blaTEM-1, blaCTX-M-15, ble, oqxA, qnrS1, mrx(A), tet(A), sul1, dfrA14, aph(3'')-Ib, aac(6')-Ib, aph(6)-Id, catB, dfrA7, oqxB32 |
| SKIMSkp30 | blaOXA-232, blaNDM-1, blaSHV-1, blaTEM-1, blaCTX-M-15, blaCMY-6, ble, oqxA, oqxB, qnrS1, estX-3, aadA1, mrx(A), mph(A), rmtC, tet(A), aadA2, dfrA12, erm(B), catB, catA1, rmtF1 |
| SKIMSkp31 | fosA, blaOXA-232, blaNDM-1, blaSHV-1, blaTEM-1, blaCTX-M-15, blaCMY-6, ble, oqxA, oqxB, qnrS1, estX-3, aadA1, mrx(A), mph(A), rmtC, tet(A), sul1, aadA2, aac(6')-Ib3, dfrA14, dfrA12, erm(B), catB, catA1, rmtF1, qnrB1 |
| SKIMSkp32 | fosA, blaOXA-232, blaSHV, blaCTX-M-15, oqxA, aadA2, oqxB25, dfrA12 |
| SKIMSkp33 | fosA, sul2, blaOXA-232, blaSHV, blaTEM-1, blaCTX-M-15, oqxA, qnrS1, mrx(A), mph(A), sul1, dfrA14, aph(3'')-Ib, aac(6')-Ib, aph(6)-Id, catB, catA1, rmtF, oqxB32 |
| SKIMSkp34 | fosA, sul2, blaOXA-1, blaOXA-232, blaSHV-1, blaTEM-1, blaCTX-M-15, blaACT-15, oqxA, oqxB, qnrS1, mrx(A), mph(A), tet(A), sul1, aadA2, dfrA14, aph(3'')-Ib, dfrA12, aph(6)-Id, erm(B), catB, catA1, rmtF1, catB3, qnrB1, aac(6')-Ib-cr5, aac(3)-IIe |
| SKIMSkp35 | fosA, blaOXA-9, blaOXA-232, blaNDM, blaSHV-1, blaTEM-1, blaCTX-M-15, ble, oqxA, oqxB, qnrS1, aadA1, mrx(A), mph(A), sul1, aadA2, dfrA14, dfrA12, catB, rmtF, aac(6')-Ib4, estX |
| SKIMSkp36 | fosA, blaOXA-232, blaNDM-1, blaSHV-1, blaTEM-1, blaCTX-M-15, blaCMY-6, ble, oqxA, estX-3, mrx(A), mph(A), rmtC, tet(A), sul1, dfrA14, aac(6')-Ib, dfrA12, erm(B), catB, catA1, arr-2, qnrS |
| SKIMSkp37 | fosA, blaOXA-232, blaSHV-1, blaTEM-1, blaCTX-M, oqxA, qnrS1, mrx(A), mph(A), sul1, aadA2, dfrA14, aac(6')-Ib, dfrA12, qnrS2, erm(B), catB, catA1, arr-2, rmtF |
| SKIMSkp38 | fosA, blaOXA-232, blaSHV-12, blaTEM-1, blaCTX-M-15, oqxA, oqxB, qnrS1, mrx(A), mph(A), sul1, aadA2, dfrA12, erm(B), catB, catA1, rmtF1 |
| SKIMSkp39 | fosA, blaOXA-232, blaNDM-1, blaSHV-12, blaTEM-1, blaCTX-M-15, blaCMY-6, ble, oqxA, oqxB, qnrS1, mrx(A), mph(A), rmtC, tet(A), sul1, aadA2, aac(6')-Ib, dfrA12, erm(B), catB, catA1, arr-2, rmtF1 |
| SKIMSkp40 | fosA, sul2, blaOXA-232, blaSHV-1, blaTEM-1, blaCTX-M-15, oqxA, oqxB, qnrS1, mrx(A), mph(A), sul1, aadA2, dfrA14, aph(3'')-Ib, dfrA12, qnrS2, aph(6)-Id, erm(B), catB, catA1, rmtF1 |
| SKIMSkp41 | fosA, blaOXA-1, blaOXA-232, blaNDM-5, blaSHV-5, blaTEM-1, blaCTX-M-15, ble, oqxA, oqxB, qnrS1, mrx(A), mph(A), sul1, aadA2, aac(6')-Ib, dfrA12, erm(B), catB, catA1, arr-2, rmtF1, catB3, qnrB1 |
| SKIMSkp42 | fosA, blaOXA-232, blaSHV-1, blaTEM-1, blaCTX-M-15, oqxA, oqxB, qnrS1, sul1, aadA2, dfrA14, erm(B), catB, catA1, rmtF1 |
| SKIMSkp43 | fosA, blaOXA-232, blaSHV, blaCTX-M-15, blaACT-15, oqxA, oqxB, qnrS1, mrx(A), mph(A), tet(A), sul1, aadA2, dfrA14, dfrA12, erm(B), catB, catA1, rmtF, qnrB1, aac(3)-IIe |
| SKIMSkp44 | fosA, blaOXA-232, blaSHV-1, blaTEM-1, blaCTX-M-15, oqxA, oqxB, qnrS1, sul1, dfrA14, erm(B), catB, catA1, arr-2, rmtF1, aac(6')-Ib4 |
| SKIMSkp45 | fosA, blaSHV-1, oqxA, qnrS1, dfrA14, oqxB32 |
| SKIMSkp46 | fosA, blaOXA, blaOXA-232, blaSHV-1, blaTEM-1, blaCTX-M-15, oqxA, qnrS1, sul1, aadA2, dfrA14, aac(6')-Ib, rmtF, oqxB32 |
| SKIMSkp47 | fosA, blaOXA-181, blaNDM-1, blaSHV-1, blaTEM-1, blaCTX-M-15, blaCMY-6, ble, oqxA, qnrS1, estX-3, aadA1, mrx(A), mph(A), rmtC, tet(A), sul1, aadA2, dfrA14, aac(6')-Ib, arr-2 |
| SKIMSkp48 | fosA, blaOXA-232, blaSHV-1, blaTEM-1, blaCTX-M-15, oqxA, oqxB, qnrS1, mrx(A), mph(A), sul1, aadA2, dfrA12, erm(B), catB, catA1, rmtF1 |
| SKIMSkp49 | fosA, sul2, blaOXA-1, blaOXA-232, blaSHV-1, blaTEM-1, blaCTX-M-15, oqxA, oqxB, qnrS1, mrx(A), mph(A), tet(A), sul1, aadA2, dfrA14, aph(3'')-Ib, dfrA12, aph(6)-Id, catB, catA1, rmtF1, catB3, qnrB1, aac(6')-Ib-cr5 |
| SKIMSkp50 | fosA, sul2, blaOXA-232, blaSHV-1, blaTEM-1, blaCTX-M-15, oqxA, oqxB, qnrS1, mrx(A), mph(A), aadA2, dfrA14, aph(3'')-Ib, aac(6')-Ib, dfrA12, aph(6)-Id, erm(B), catB, catA1, arr-2 |
| SKIMSkp51 | fosA, sul2, blaOXA-232, blaSHV-1, blaTEM-1, blaCTX-M-15, oqxA, oqxB, qnrS1, sul1, aadA2, dfrA14, aph(3'')-Ib, dfrA12, catB, catA1, rmtF1, qnrB1 |
| SKIMSkp52 | fosA, blaOXA-232, blaSHV-1, blaTEM-1, blaCTX-M-15, oqxA, qnrS1, dfrA14, aac(6')-Ib, catA1, arr-2, rmtF, oqxB32 |
| SKIMSkp53 | fosA, blaOXA-232, blaNDM-5, blaSHV-1, blaTEM, blaCTX-M-15, ble, oqxA, qnrS1, mrx(A), mph(A), sul1, aadA2, dfrA14, dfrA12, erm(B), catB, catA1, rmtF1, oqxB19, dfrA50, qnrB |
| SKIMSkp54 | fosA, blaOXA-1, blaOXA-232, blaSHV-1, blaTEM-1, blaCTX-M-15, oqxA, oqxB, qnrS1, sul1, aadA2, dfrA14, aac(6')-Ib, dfrA12, catB, arr-2, catB3, armA, mph(E), msr(E) |
| SKIMSkp55 | fosA, blaOXA, blaOXA-232, blaSHV-1, blaCTX-M-15, oqxA, oqxB, qnrS1, mrx(A), mph(A), sul1, aadA2, dfrA14, aac(6')-Ib, dfrA12, erm(B), catB, catA1, arr-2, catB3 |
| SKIMSkp56 | blaOXA-232, blaSHV, blaCTX-M, qnrS1, mrx(A), mph(A), sul1, aadA2, dfrA14, aac(6')-Ib, dfrA12, erm(B), catB, catA1, arr-2, rmtF |
| SKIMSkp57 | fosA, blaOXA-232, blaSHV-1, blaTEM-1, blaCTX-M-15, blaCMY-6, oqxA, qnrS1, mrx(A), mph(A), rmtC, tet(A), sul1, aadA2, aac(6')-Ib3, dfrA14, arr-2, oqxB32 |
| SKIMSkp58 | fosA, blaOXA-232, blaSHV-1, blaCTX-M-15, oqxA, qnrS1, sul1, aadA2, dfrA14, rmtF1, oqxB32 |
| SKIMSkp59 | fosA, blaOXA-1, blaOXA-232, blaNDM-1, blaSHV-1, blaTEM-1, blaCTX-M-15, blaCMY-6, ble, oqxA, qnrS1, estX-3, aadA1, mrx(A), mph(A), rmtC, tet(A), sul1, aadA2, aac(6')-Ib3, qnrS2, erm(B), catB, catA1, arr-2, rmtF, catB3, qnrB1, oqxB20, tet(D), aph(3')-Ia |
| SKIMSkp60 | fosA, blaSHV-1, oqxA, qnrS1, sul1, dfrA14, arr-2, oqxB32 |
| SKIMSkp61 | fosA, blaOXA-232, blaSHV-1, oqxA, qnrS1, dfrA14, oqxB32 |
| SKIMSkp62 | fosA, sul2, blaOXA-232, blaSHV-1, blaTEM-1, blaCTX-M-15, oqxA, oqxB, qnrS1, mrx(A), mph(A), sul1, aadA2, dfrA14, aph(3'')-Ib, aac(6')-Ib, dfrA12, qnrS2, aph(6)-Id, erm(B), catB, catA1, arr-2 |
| SKIMSkp63 | fosA, blaOXA-232, blaSHV-1, oqxA, qnrS1, dfrA14, oqxB32 |
| SKIMSkp64 | fosA, sul2, blaOXA-232, blaSHV-1, blaTEM, blaCTX-M-15, oqxA, qnrS1, dfrA14, aph(3'')-Ib, aph(6)-Id, catB, catA1, oqxB32 |
| SKIMSkp65 | fosA, blaOXA-232, blaSHV-1, blaTEM-1, blaCTX-M-15, qnrS1, mrx(A), mph(A), sul1, aadA2, dfrA14, dfrA12, catB, catA1, rmtF1 |
| SKIMSkp66 | fosA, blaOXA-232, blaNDM, blaSHV-1, blaCTX-M-15, oqxA, oqxB, qnrS1, mrx(A), mph(A), tet(A), sul1, aadA2, dfrA14, estX, aph(3')-Ia |
| SKIMSkp67 | fosA, blaOXA, blaSHV-1, blaTEM-1, blaCTX-M-15, oqxA, mrx(A), mph(A), tet(A), sul1, aadA2, dfrA14, aac(6')-Ib, dfrA12, erm(B), catB, catA1, arr-2, catB3, oqxB19, aph(3')-Ia |
| SKIMSkp68 | fosA, blaOXA-232, blaNDM-5, blaSHV-1, blaTEM-1, blaCTX-M-15, ble, oqxA, mrx(A), mph(A), tet(A), sul1, aadA2, dfrA14, aac(6')-Ib, dfrA12, catB, catA1, arr-2, qnrS, rmtF, qnrB |
| SKIMSkp69 | fosA, blaOXA-232, blaSHV-1, blaCTX-M-15, oqxA, oqxB, qnrS1, dfrA14, qnrB1 |
| SKIMSkp70 | fosA, sul2, blaOXA-1, blaOXA-9, blaOXA-232, blaNDM-1, blaSHV-27, blaTEM-1, blaCTX-M-15, ble, oqxA, qnrS1, aadA1, tet(A), sul1, dfrA14, aph(3'')-Ib, aph(6)-Id, arr-2, catB3, qnrB1, aac(3)-IIe, oqxB19, dfrA1 |
| SKIMSkp71 | fosA, sul2, blaOXA-9, blaOXA-232, blaNDM-5, blaSHV-1, blaTEM, blaCTX-M-15, ble, oqxA, qnrS1, aadA1, mrx(A), mph(A), sul1, dfrA14, aph(3'')-Ib, aac(6')-Ib, aph(6)-Id, rmtB1, oqxB32, tet(B), dfrA1, arr-3 |
| SKIMSkp72 | fosA, sul2, blaOXA-9, blaOXA-232, blaNDM-5, blaSHV-1, blaTEM, blaCTX-M-15, ble, oqxA, qnrS1, aadA1, mrx(A), mph(A), sul1, dfrA14, aph(3'')-Ib, aac(6')-Ib, aph(6)-Id, erm(B), rmtB1, oqxB32, tet(B), dfrA1, arr-3 |
| SKIMSkp73 | fosA, sul2, blaOXA-232, blaSHV-1, blaTEM-1, blaCTX-M-15, oqxA, oqxB, qnrS1, mrx(A), mph(A), sul1, aadA2, dfrA14, aph(3'')-Ib, dfrA12, aph(6)-Id, erm(B), catB, catA1, rmtF1 |
| SKIMSkp74 | fosA, sul2, blaOXA-232, blaSHV-1, blaTEM-1, blaCTX-M-15, oqxA, oqxB, qnrS1, mrx(A), mph(A), sul1, aadA2, dfrA14, aph(3'')-Ib, dfrA12, aph(6)-Id, erm(B), catB, catA1, rmtF1 |
| SKIMSkp75 | fosA, blaOXA-1, blaOXA-232, blaNDM-1, blaSHV-1, blaTEM-1, blaCTX-M, ble, oqxA, oqxB, qnrS1, estX-3, aadA1, mrx(A), mph(A), tet(A), sul1, aadA2, aac(6')-Ib, dfrA12, erm(B), catB, catA1, arr-2, rmtF, catB3, aph(3')-Ia, aph(3')-VI |
| SKIMSkp76 | fosA, blaOXA-232, blaSHV-1, blaCTX-M-15, oqxA, oqxB, qnrS1, mrx(A), mph(A), sul1, aadA2, dfrA12, erm(B), catB, catA1, rmtF1 |
| SKIMSkp77 | fosA, blaOXA-232, blaSHV-1, blaCTX-M-15, oqxA, qnrS1, sul1, aadA2, dfrA14, dfrA12, catA1, rmtF1, oqxB32 |
| SKIMSkp78 | fosA, sul2, blaOXA-1, blaNDM-5, blaSHV-1, blaTEM-1, blaCTX-M-15, ble, oqxA, oqxB, qnrS1, aadA1, mrx(A), mph(A), tet(A), sul1, aadA2, dfrA14, aph(3'')-Ib, dfrA12, aph(6)-Id, erm(B), catB, catA1, arr-2, rmtB1, rmtF1, catB3, qnrB1, aac(6')-Ib-cr5, aac(3)-IIe, cmlA5, ere(A) |
| SKIMSkp79 | fosA, sul2, blaOXA-1, blaOXA-232, blaSHV-1, blaTEM-1, blaCTX-M-15, oqxA, oqxB, qnrS1, aadA1, mrx(A), mph(A), sul1, aadA2, aph(3'')-Ib, aac(6')-Ib, dfrA12, aph(6)-Id, erm(B), catB, catA1, rmtF1, catB3, qnrB1, cmlA5 |
| SKIMSkp80 | fosA, sul2, blaOXA-1, blaSHV-27, blaTEM-1, blaCTX-M-15, oqxA, qnrS1, tet(A), dfrA14, aph(3'')-Ib, aph(6)-Id, catB3, qnrB1, aac(6')-Ib-cr5, aac(3)-IIe |
| SKIMSkp81 | fosA, sul2, blaOXA-232, blaSHV-1, blaTEM-1, blaCTX-M-15, oqxA, oqxB, qnrS1, dfrA14, aph(3'')-Ib, aph(6)-Id |
| SKIMSkp82 | fosA, sul2, blaOXA-9, blaOXA-232, blaNDM-5, blaSHV-1, blaTEM, blaCTX-M-15, ble, oqxA, qnrS1, aadA1, mrx(A), mph(A), sul1, dfrA14, aph(3'')-Ib, aac(6')-Ib, aph(6)-Id, erm(B), rmtB1, oqxB32, tet(B), dfrA1, arr-3 |
| SKIMSkp83 | fosA, sul2, blaOXA-232, blaSHV-1, blaTEM-1, blaCTX-M-15, oqxA, oqxB, qnrS1, mrx(A), sul1, aadA2, dfrA14, aph(3'')-Ib, aac(6')-Ib, dfrA12, aph(6)-Id, erm(B), catB, catA1 |
| SKIMSkp84 | fosA, sul2, blaOXA-232, blaSHV-33, blaTEM, blaCTX-M-15, oqxA, oqxB, qnrS1, dfrA14, aph(3'')-Ib, aac(6')-Ib, aph(6)-Id, rmtF |
| SKIMSkp85 | blaOXA-232, blaSHV, blaTEM-1, blaCTX-M-15, qnrS1, dfrA14, aac(6')-Ib, catB, arr-2 |
| SKIMSkp86 | fosA, sul2, blaOXA-232, blaSHV-1, blaTEM-1, blaCTX-M-15, oqxA, qnrS1, mrx(A), mph(A), sul1, aadA2, dfrA14, aph(3'')-Ib, dfrA12, aph(6)-Id, catB, catA1, rmtF1, oqxB32 |
| SKIMSkp87 | fosA, sul2, blaOXA-1, blaOXA-232, blaSHV-1, blaTEM-1, blaCTX-M-15, oqxA, qnrS1, dfrA14, aph(3'')-Ib, qnrS2, aph(6)-Id, catB3, oqxB32, aac(6')-Ib-cr5, qnrB |
| SKIMSkp88 | fosA, sul2, blaOXA-1, blaOXA-232, blaSHV-1, blaTEM-1, blaCTX-M-15, oqxA, qnrS1, tet(A), dfrA14, aph(3'')-Ib, aph(6)-Id, catB3, qnrB1, aac(6')-Ib-cr5, aac(3)-IIe, oqxB19 |
| SKIMSkp89 | fosA, sul2, blaOXA-1, blaSHV-11, blaTEM-1, blaCTX-M-15, oqxA, oqxB, tet(A), dfrA14, aph(3'')-Ib, aph(6)-Id, catB3, qnrB1, aac(6')-Ib-cr5, aac(3)-IIe |
| SKIMSkp90 | fosA, blaSHV-11, oqxA, oqxB |
| SKIMSkp91 | sul2, blaTEM-1, blaCTX-M-15, qnrS1, dfrA14, aph(3'')-Ib, aph(6)-Id |
| SKIMSkp92 | fosA, sul2, blaOXA-232, blaSHV, blaTEM-1, blaCTX-M, oqxA, qnrS1, mrx(A), mph(A), sul1, aadA2, dfrA14, aph(3'')-Ib, aac(6')-Ib, dfrA12, aph(6)-Id, erm(B), catB, catA1, arr-2, rmtF |
| SKIMSkp93 | fosA, sul2, blaOXA, blaOXA-232, blaSHV-1, blaTEM-1, blaCTX-M-15, oqxA, oqxB, qnrS1, mrx(A), mph(A), sul1, aadA2, dfrA14, aph(3'')-Ib, aac(6')-Ib, dfrA12, aph(6)-Id, erm(B), catB, catA1, arr-2, catB3, qnrB1 |
| SKIMSkp94 | sul2, blaTEM-1, blaCTX-M-15, qnrS1, dfrA14, aph(3'')-Ib, aph(6)-Id |
| SKIMSkp95 | sul2, blaSHV, blaTEM-1, blaCTX-M-15, oqxB, qnrS1, dfrA14, aph(3'')-Ib, aph(6)-Id |
| SKIMSkp96 | fosA, blaOXA-1, blaOXA-232, blaSHV-33, blaTEM-1, blaCTX-M-15, blaDHA-1, oqxB, qnrS1, estX-3, mrx(A), mph(A), sul1, aadA2, aac(6')-Ib, cmlA1, sul3, catB3, aph(3')-Ia, arr-3, oqxA3, qnrB4, dfrA17 |
