# Supplementary material for: Comprehensive genomic characterization of multidrug-resistant Klebsiella pneumoniae from North India
Source: Front Microbiol. 2026 Sep 24;17:1831365. doi: 10.3389/fmicb.2026.1831365 (PMC13646146; doi:10.3389/fmicb.2026.1831365)
Supplement: Supplementary file 4 [file Table_4.DOCX]

| **Supplementary Table 4. Distribution of Resistance-associated point mutations (RAPM) with associated genes in *K. pneumoniae* study isolates** | |
| --- | --- |
| **Isolates** | **Resistance-associated point mutations with associated genes** |
| SKIMSkp02 | gyrA_D87G, gyrA_S83I, ompK36_T136TDT, ompK35_Y108TerfsTer0, parC_S80I |
| SKIMSkp11 | parC_S80I, gyrA_D87A, gyrA_S83F, ramR_A19V |
| SKIMSkp16 | gyrA_D87A, gyrA_S83F |
| SKIMSkp19 | ompK36_T136TDT, gyrA_S83I, cirA_N327IfsTer4, parC_S80I, ompK35_Y108TerfsTer0 |
| SKIMSkp28 | ompK35_Y108TerfsTer0 |
| SKIMSkp30 | parC_S80I, ompK35_Y108TerfsTer0, gyrA_S83I |
| SKIMSkp31 | ompK35_Y108TerfsTer0, ramR_A19V |
| SKIMSkp34 | oqxR_V113I |
| SKIMSkp36 | parC_S80I, gyrA_S83I |
| SKIMSkp38 | gyrA_S83T, gyrA_S83I, parC_S80I |
| SKIMSkp40 | ompK35_Y108TerfsTer0, gyrA_S83I, ompK36_T136TDT |
| SKIMSkp43 | oqxR_V113I |
| SKIMSkp47 | gyrA_D87A, gyrA_S83F |
| SKIMSkp48 | parC_S80I |
| SKIMSkp53 | parC_S80I, gyrA_S83T |
| SKIMSkp54 | gyrA_S83T |
| SKIMSkp59 | parC_S80I, gyrA_S83T |
| SKIMSkp71 | nfsB_V167GfsTer7, cirA_T54SfsTer13, ompK36_D135DD |
| SKIMSkp75 | ompK35_Y108TerfsTer0, gyrA_S83I, parC_S80I, ompK36_T136TDT |
| SKIMSkp76 | ompK35_Y108TerfsTer0, gyrA_S83I, ompK36_T136TDT, parC_S80I |
| SKIMSkp82 | cirA_T54SfsTer13, ompK36_D135DD, nfsB_V167GfsTer7 |
| SKIMSkp89 | gyrA_S83T |
| SKIMSkp92 | gyrA_S83I, ompK36_T136TDT |
