# Supplementary material for: Comprehensive genomic characterization of multidrug-resistant Klebsiella pneumoniae from North India
Source: Front Microbiol. 2026 Sep 24;17:1831365. doi: 10.3389/fmicb.2026.1831365 (PMC13646146; doi:10.3389/fmicb.2026.1831365)
Supplement: Supplementary file 5 [file Table_5.DOCX]

| **Supplementary Table 5. Distribution of Plasmids replicons in *K. pneumoniae* study isolates** | |
| --- | --- |
| **Isolates** | **Plasmids** |
| SKIMSkp01 | Col440I, IncFII(pKP91), IncHI1B(pNDM-MAR), IncX3, repFIB |
| SKIMSkp02 | Col440I, ColKP3, IncFIA, IncFIB(AP001918), IncFIB(K)(pCAV1099-114), IncFIB(pQil), IncFII(K), IncFII(pAMA1167-NDM-5), IncFII(pRSB107), IncHI1B(pNDM-MAR), IncX3, repFIB |
| SKIMSkp03 | Col440I, IncFIB(K)(pCAV1099-114), IncHI1B(pNDM-MAR), IncX3, repFIB |
| SKIMSkp04 | Col440I, IncFIB(K)(pCAV1099-114), IncHI1B(pNDM-MAR), IncX3, repFIB |
| SKIMSkp05 | Col440I, IncFIB(K)(pCAV1099-114), IncHI1B(pNDM-MAR), IncN, IncX3, RepB, repFIB |
| SKIMSkp06 | Col(pHAD28), IncFIB(K)(pCAV1099-114), IncHI1B(pNDM-MAR), IncX3, repFIB |
| SKIMSkp07 | Col440I, Col440II, ColKP3, ColpVC, IncFIB(K), IncFIB(pQil), IncFII(K), IncFII(pAMA1167-NDM-5), IncFII(pKP91), IncHI1B(pNDM-MAR), IncX3, RepB, repFIB |
| SKIMSkp08 | Col(pHAD28), IncFIB(K), IncFII(pKP91) |
| SKIMSkp09 | Col440II, ColKP3, IncFIA, IncFIB(K)(pCAV1099-114), IncFIB(pQil), IncFII(K), IncHI1B(pNDM-MAR), IncX3, RepB, repFIB |
| SKIMSkp11 | Col440I, Col440II, ColKP3, IncC, IncFIB(K), IncFII(K), IncX3 |
| SKIMSkp12 | Col(pHAD28), Col440I, Col440II, IncFIB(K)(pCAV1099-114), IncHI1B(pNDM-MAR), IncX3, RepB, repFIB |
| SKIMSkp13 | Col440I, Col440II, IncFIB(K)(pCAV1099-114), IncHI1B(pNDM-MAR), IncX3, RepB, repFIB |
| SKIMSkp14 | Col440I, Col440II, IncFIB(K)(pCAV1099-114), IncFII(pAMA1167-NDM-5), IncHI1B(pNDM-MAR), IncX3, repFIB |
| SKIMSkp15 | Col440I, Col440II, ColKP3, IncC, IncFIB(K), IncFIB(K)(pCAV1099-114), IncFII(K), IncHI1B(pNDM-MAR), IncQ2, IncX3, repFIB |
| SKIMSkp16 | Col440I, Col440II, ColKP3, IncC, IncFIB(K), IncFIB(K)(pCAV1099-114), IncFII(K), IncX3, RepB |
| SKIMSkp17 | ColKP3, ColpVC, IncFIA, IncFIB(pQil), IncFII(pAMA1167-NDM-5), IncFII(pRSB107), IncHI1B(pNDM-MAR), IncX3, RepB |
| SKIMSkp18 | ColKP3, IncFIB(K)(pCAV1099-114), IncFIB(pQil), IncFII(K), RepB, repFIB |
| SKIMSkp19 | Col(pHAD28), Col440I, Col440II, ColKP3, IncFIB(K), IncFIB(pQil), IncFII, IncFII(K), IncN4, IncX3 |
| SKIMSkp20 | Col440I, IncFIA(HI1), IncFIB(K)(pCAV1099-114), IncFII(pSDP9R), IncHI1B(pNDM-MAR), IncR, IncX3, repFIB |
| SKIMSkp21 | ColKP3, IncFIB(K)(pCAV1099-114), IncFIB(pQil), IncFII(K), IncFII(pRSB107), IncHI1B(pNDM-MAR), IncN, RepB, repFIB |
| SKIMSkp22 | Col440I, ColKP3, IncFIB(K)(pCAV1099-114), IncFII(pKP91), IncHI1B(pNDM-MAR), IncN, IncX3, RepB, repFIB |
| SKIMSkp23 | Col440I, Col440II, ColKP3, IncFIA, IncFIB(K)(pCAV1099-114), IncFIB(pQil), IncFII(K), IncFII(pAMA1167-NDM-5), IncFII(pRSB107), IncHI1B(pNDM-MAR), IncX3, RepB, repFIB |
| SKIMSkp24 | Col440I, Col440II, ColKP3, IncFIB(K)(pCAV1099-114), IncHI1B(pNDM-MAR), IncQ2, IncX3, repFIB |
| SKIMSkp25 | Col440II, ColKP3, ColpVC, IncC, IncFIA, IncFIB(K)(pCAV1099-114), IncFIB(pQil), IncFII(K), IncHI1B(pNDM-MAR), IncQ2, IncX3, repFIB |
| SKIMSkp26 | Col440I, ColKP3, IncFIB(K)(pCAV1099-114), IncFIB(pQil), IncFII(pAMA1167-NDM-5), IncFII(pRSB107), IncHI1B(pNDM-MAR), IncX3, RepB, repFIB |
| SKIMSkp27 | Col440II, ColKP3, ColRNAI, ColpVC, IncC, IncFIA(pBK30683), IncFII(pKP91), IncHI1B(pNDM-MAR), IncX3, RepB, repFIB |
| SKIMSkp28 | Col(pHAD28), Col440I, Col440II, ColKP3, IncC, IncFIA, IncFIB(K)(pCAV1099-114), IncFIB(pQil), IncFII(K), IncFII(pAMA1167-NDM-5), IncFII(pRSB107), IncHI1B(pNDM-MAR), IncX3, repFIB |
| SKIMSkp29 | Col440I, Col440II, ColpVC, IncFII(K), IncFII(pKP91), IncHI1B(pNDM-MAR), IncX3, RepB, repFIB |
| SKIMSkp30 | Col(pHAD28), Col440I, Col440II, ColKP3, IncC, IncFIA, IncFIB(K), IncFIB(K)(pCAV1099-114), IncFIB(pQil), IncFII(K), IncFII(pAMA1167-NDM-5), IncFII(pRSB107), IncX3, RepB |
| SKIMSkp31 | Col(pHAD28), Col440I, Col440II, ColKP3, IncC, IncFIA, IncFIB(K), IncFIB(K)(pCAV1099-114), IncFIB(pQil), IncFII(K), IncFII(pAMA1167-NDM-5), IncFII(pRSB107), IncHI1B(pNDM-MAR), IncX3, RepB |
| SKIMSkp32 | Col440I, ColKP3, IncFIB(K)(pCAV1099-114), IncHI1B(pNDM-MAR), IncX3, repFIB |
| SKIMSkp33 | Col440I, Col440II, ColKP3, IncFIA, IncFIB(K)(pCAV1099-114), IncFIB(pQil), IncFII(K), IncHI1B(pNDM-MAR), IncR, IncX3, IncY, RepB, repFIB |
| SKIMSkp34 | Col(pHAD28), Col440I, Col440II, ColKP3, IncFIA, IncFIB(K)(pCAV1099-114), IncFIB(pQil), IncFII(K), IncFII(pAMA1167-NDM-5), IncFII(pRSB107), IncHI1B(pNDM-MAR), IncN, IncX3, RepB, repFIB |
| SKIMSkp35 | Col(pHAD28), Col440I, Col440II, ColKP3, IncFIA, IncFIB(K)(pCAV1099-114), IncFIB(pQil), IncFII(K), IncHI1B(pNDM-MAR), IncN, IncX3, RepB |
| SKIMSkp36 | Col440II, ColKP3, IncC, IncFIA, IncFIB(K), IncFIB(pQil), IncFII(K), IncFII(pAMA1167-NDM-5), IncFII(pRSB107), IncHI1B(pNDM-MAR), IncQ2, IncX3, repFIB |
| SKIMSkp37 | Col440I, Col440II, ColKP3, IncFIA, IncFIB(K)(pCAV1099-114), IncFIB(pQil), IncFII(K), IncFII(pAMA1167-NDM-5), IncFII(pRSB107), IncN, IncQ2, RepB |
| SKIMSkp38 | Col440I, Col440II, ColKP3, IncFIA, IncFIB(K)(pCAV1099-114), IncFIB(pQil), IncFII(K), IncFII(pAMA1167-NDM-5), IncFII(pRSB107), IncHI1B(pNDM-MAR), IncN, IncX3, RepB |
| SKIMSkp39 | Col440I, Col440II, ColKP3, ColpVC, IncC, IncFIA, IncFIB(K), IncFIB(K)(pCAV1099-114), IncFIB(pQil), IncFII(K), IncFII(pAMA1167-NDM-5), IncFII(pRSB107), IncHI1B(pNDM-MAR), IncX3, repFIB |
| SKIMSkp40 | Col(pHAD28), Col440I, Col440II, ColKP3, ColpVC, IncFIA, IncFIB(K)(pCAV1099-114), IncFIB(pQil), IncFII(K), IncFII(pAMA1167-NDM-5), IncFII(pRSB107), IncQ2, IncX3, RepB, repFIB |
| SKIMSkp41 | Col440I, Col440II, ColKP3, ColpVC, IncFIA, IncFIA(pBK30683), IncFIB(K)(pCAV1099-114), IncFIB(pQil), IncFII(K), IncFII(pAMA1167-NDM-5), IncFII(pRSB107), IncHI1B(pNDM-MAR), IncN, IncX3, RepB, repFIB |
| SKIMSkp42 | Col440I, ColKP3, IncFIB(K)(pCAV1099-114), IncFIB(pQil), IncFII(pRSB107), IncN, RepB |
| SKIMSkp43 | Col(pHAD28), Col440I, ColKP3, IncFIA, IncFIB(pQil), IncFII(K), IncFII(pAMA1167-NDM-5), IncHI1B(pNDM-MAR), IncN, IncX3, RepB, repFIB |
| SKIMSkp44 | Col440I, Col440II, ColKP3, ColRNAI, IncFIA, IncFIB(K)(pCAV1099-114), IncFIB(pQil), IncFII(K), IncFII(pAMA1167-NDM-5), IncN, IncX3, RepB |
| SKIMSkp45 | IncFIB(K)(pCAV1099-114), IncN, RepB |
| SKIMSkp46 | Col(pHAD28), Col440I, ColKP3, ColpVC, IncFIB(K)(pCAV1099-114), IncN, RepB |
| SKIMSkp47 | Col440I, Col440II, ColKP3, ColpVC, IncC, IncFIB(K), IncFIB(K)(pCAV1099-114), IncFII(K), IncN, IncX3, RepB |
| SKIMSkp48 | Col(pHAD28), Col440I, ColKP3, ColpVC, IncFIA, IncFIB(K)(pCAV1099-114), IncFIB(pQil), IncFII, IncFII(K), IncN, RepB |
| SKIMSkp49 | Col(pHAD28), Col440I, ColKP3, ColpVC, IncFIA, IncFIA(pBK30683), IncFIB(K)(pCAV1099-114), IncFIB(pQil), IncFII(K), IncFII(pAMA1167-NDM-5), IncN, RepB |
| SKIMSkp50 | Col440I, ColKP3, IncFIA, IncFIB(K)(pCAV1099-114), IncFIB(pQil), IncFII(K), IncN, RepB |
| SKIMSkp51 | Col(pHAD28), Col440I, ColKP3, ColpVC, IncFIA, IncFIB(K)(pCAV1099-114), IncFIB(pQil), IncFII(K), IncFII(pAMA1167-NDM-5), IncFII(pRSB107), IncN, RepB |
| SKIMSkp52 | Col440I, ColKP3, IncFIB(K)(pCAV1099-114), IncFIB(pQil), IncFII(K), IncN, RepB |
| SKIMSkp53 | Col440I, ColKP3, ColRNAI, ColpVC, IncFIA, IncFIB(K)(pCAV1099-114), IncFIB(pQil), IncFII, IncFII(K), IncFII(pAMA1167-NDM-5), IncFII(pRSB107), IncN, RepB, repB(R1701) |
| SKIMSkp54 | Col(pHAD28), Col440I, ColKP3, ColRNAI, IncFIA, IncFIB(K)(pCAV1099-114), IncFIB(pNDM-Mar), IncFIB(pQil), IncFII(K), IncHI1B(pNDM-MAR), IncI1-I(Alpha), IncN, RepB |
| SKIMSkp55 | ColKP3, ColpVC, IncFIA, IncFIB(K)(pCAV1099-114), IncFIB(pQil), IncFII(K), IncFII(pAMA1167-NDM-5), IncFII(pRSB107), IncN, RepB |
| SKIMSkp56 | Col440I, ColKP3, IncFIA, IncFIB(K)(pCAV1099-114), IncFIB(pQil), IncFII(K), IncFII(pAMA1167-NDM-5), IncFII(pRSB107), IncN, RepB |
| SKIMSkp57 | Col440I, Col440II, ColKP3, ColpVC, IncC, IncFIB(K)(pCAV1099-114), IncFII(K), IncFII(pAMA1167-NDM-5), IncN, IncX3, RepB |
| SKIMSkp58 | Col(pHAD28), Col440I, Col440II, ColKP3, ColpVC, IncFIB(K)(pCAV1099-114), IncN, IncX3, RepB |
| SKIMSkp59 | Col(pHAD28), Col440I, Col440II, ColKP3, ColpVC, IncC, IncFIA, IncFIB(K), IncFIB(K)(pCAV1099-114), IncFIB(pQil), IncFII(K), IncFII(pAMA1167-NDM-5), IncQ2, IncX3, RepB |
| SKIMSkp60 | Col440I, Col440II, IncFIB(K)(pCAV1099-114), IncN, RepB |
| SKIMSkp61 | Col(pHAD28), ColKP3, IncFIB(K)(pCAV1099-114), IncN, RepB |
| SKIMSkp62 | Col440I, Col440II, ColKP3, IncFIA, IncFIB(K)(pCAV1099-114), IncFIB(pQil), IncFII(K), IncFII(pAMA1167-NDM-5), IncFII(pRSB107), IncHI1B(pNDM-MAR), IncN, IncQ2, RepB, repFIB |
| SKIMSkp63 | Col440I, ColKP3, IncFIB(K)(pCAV1099-114), IncN, RepB |
| SKIMSkp64 | Col(pHAD28), Col440I, ColKP3, IncFIB(K)(pCAV1099-114), IncFIB(pQil), IncN, RepB |
| SKIMSkp65 | Col(pHAD28), Col440I, ColKP3, ColRNAI, IncFIA, IncFIB(K)(pCAV1099-114), IncFIB(pQil), IncFII(K), IncN, RepB |
| SKIMSkp66 | Col(pHAD28), Col440I, Col440II, ColKP3, IncFIB(K)(pCAV1099-114), IncFII(pAMA1167-NDM-5), IncN, RepB |
| SKIMSkp67 | IncFIA, IncFIB(K)(pCAV1099-114), IncFIB(pQil), IncFII(pAMA1167-NDM-5), IncFII(pRSB107), IncN, RepB |
| SKIMSkp68 | ColKP3, ColpVC, IncFIA, IncFIB(K)(pCAV1099-114), IncFIB(pQil), IncFII(K), IncN, IncQ2, RepB |
| SKIMSkp69 | Col440I, ColKP3, IncFIB(K), IncFIB(K)(pCAV1099-114), IncFII(pEH01), IncFII(pKP91), IncN, RepB |
| SKIMSkp70 | Col(pHAD28), Col440I, Col440II, ColKP3, ColRNAI, ColpVC, IncFIB(K), IncFIB(K)(pCAV1099-114), IncFIB(pQil), IncFII(K), IncN, RepB |
| SKIMSkp71 | Col440I, Col440II, ColKP3, ColpVC, IncFIA, IncFIB(AP001918), IncFIB(K)(pCAV1099-114), IncFIB(pQil), IncFII, IncFII(K), IncFII(pSE11), IncX3, RepB, repFIB |
| SKIMSkp72 | Col440I, ColKP3, ColpVC, IncFIA, IncFIA(pBK30683), IncFIB(AP001918), IncFIB(K)(pCAV1099-114), IncFIB(pQil), IncFII, IncFII(K), IncFII(pKP91), IncX3, RepB, repFIB |
| SKIMSkp73 | Col440I, ColKP3, ColRNAI, IncFIA, IncFIB(K)(pCAV1099-114), IncFIB(pQil), IncFII(K), IncFII(pAMA1167-NDM-5), IncFII(pRSB107), IncN, RepB, repFIB |
| SKIMSkp74 | Col(pHAD28), Col440I, ColKP3, ColRNAI, ColpVC, IncFIA, IncFIB(K)(pCAV1099-114), IncFIB(pQil), IncFII(K), IncFII(pAMA1167-NDM-5), IncFII(pRSB107), IncN, RepB |
| SKIMSkp75 | Col440I, Col440II, ColKP3, ColRNAI, IncFIA, IncFIB(K)(pCAV1099-114), IncFIB(pQil), IncFII(K), IncFII(pAMA1167-NDM-5), IncFII(pRSB107), IncN, IncN4, IncR, IncX3, RepB |
| SKIMSkp76 | Col(pHAD28), ColKP3, IncFIA, IncFIB(pQil), IncFII(K), IncFII(pAMA1167-NDM-5), IncFII(pRSB107) |
| SKIMSkp77 | Col440I, ColKP3, ColRNAI, IncFIB(K)(pCAV1099-114), IncFIB(pQil), IncFII(K), IncN, IncX3, RepB |
| SKIMSkp78 | Col(pHAD28), Col440I, Col440II, IncFIA, IncFIB(K), IncFIB(K)(pCAV1099-114), IncFIB(pQil), IncFII, IncFII(K), IncN, IncR, RepB, repFIB |
| SKIMSkp79 | Col(pHAD28), Col440I, Col440II, ColKP3, IncFIA, IncFIB(K)(pCAV1099-114), IncFIB(pQil), IncFII(K), IncFII(pAMA1167-NDM-5), IncFII(pRSB107), IncN, RepB, repFIB |
| SKIMSkp80 | IncFIB(K), IncFIB(K)(pCAV1099-114), IncFII(K), IncN, RepB |
| SKIMSkp81 | Col(pHAD28), Col440I, ColKP3, IncFIA(pBK30683), IncFIB(K)(pCAV1099-114), IncFII(pKP91), IncN, RepB, repFIB |
| SKIMSkp82 | Col440I, ColKP3, ColpVC, IncFIA, IncFIB(AP001918), IncFIB(K)(pCAV1099-114), IncFIB(pQil), IncFII, IncFII(K), IncFII(pSE11), IncX3, RepB, repFIB |
| SKIMSkp83 | Col(pHAD28), Col440I, ColKP3, ColpVC, IncFIB(K)(pCAV1099-114), IncFIB(pQil), IncFII(K), IncX3, RepB, repFIB |
| SKIMSkp84 | Col(pHAD28), Col440I, ColKP3, ColpVC, IncFIB(K)(pCAV1099-114), IncFII(pAMA1167-NDM-5), RepB, repFIB |
| SKIMSkp85 | ColKP3, IncFIB(K)(pCAV1099-114), IncFIB(pQil), IncN, RepB |
| SKIMSkp86 | Col(pHAD28), Col440I, ColKP3, ColpVC, IncFIB(K)(pCAV1099-114), IncFIB(pQil), IncFII(K), IncFII(pAMA1167-NDM-5), IncFII(pRSB107), IncN, RepB, repFIB |
| SKIMSkp87 | Col440I, ColKP3, IncFIB(K), IncFIB(K)(pCAV1099-114), IncHI1B(pNDM-MAR), IncN, IncQ2, RepB, repFIB |
| SKIMSkp88 | Col440I, IncFIB(K), IncFIB(K)(pCAV1099-114), IncFII(K), IncN, RepB |
| SKIMSkp89 | Col(pHAD28), IncFII(K), RepB |
| SKIMSkp90 | Col(pHAD28), RepB |
| SKIMSkp91 | Col440I, IncFIA(pBK30683), IncFIB(K)(pCAV1099-114), IncFII(pKP91), IncN, RepB, repFIB |
| SKIMSkp92 | ColKP3, ColpVC, IncFIA, IncFIB(K)(pCAV1099-114), IncFIB(pQil), IncFII(K), RepB, repFIB |
| SKIMSkp93 | Col440II, ColKP3, IncFIA(pBK30683), IncFIB(K)(pCAV1099-114), IncFIB(pQil), IncFII(K), IncX3, RepB, repFIB |
| SKIMSkp94 | Col440I, IncFIA(pBK30683), IncFIB(K)(pCAV1099-114), IncN, RepB, repFIB |
| SKIMSkp95 | Col440I, IncFIA(pBK30683), IncFIB(K)(pCAV1099-114), IncFII(pKP91), RepB, repFIB |
| SKIMSkp96 | Col440I, ColKP3, IncFIA, IncFIB(AP001918), IncFIB(K)(pCAV1099-114), IncFII(K), IncFII(pRSB107), IncFII(pSDP9R), IncR, RepB, repB(R1701) |
